# Search for physics beyond the standard model in events with two leptons of same sign, missing transverse momentum, and jets in proton–proton collisions at $\sqrt{s} = 13\,\text {TeV}$

**DOI:** 10.1140/epjc/s10052-017-5079-z

**Published:** 2017-09-01

**Authors:** A. M. Sirunyan, A. Tumasyan, W. Adam, F. Ambrogi, E. Asilar, T. Bergauer, J. Brandstetter, E. Brondolin, M. Dragicevic, J. Erö, M. Flechl, M. Friedl, R. Frühwirth, V. M. Ghete, J. Grossmann, J. Hrubec, M. Jeitler, A. König, N. Krammer, I. Krätschmer, D. Liko, T. Madlener, I. Mikulec, E. Pree, D. Rabady, N. Rad, H. Rohringer, J. Schieck, R. Schöfbeck, M. Spanring, D. Spitzbart, J. Strauss, W. Waltenberger, J. Wittmann, C. -E. Wulz, M. Zarucki, V. Chekhovsky, V. Mossolov, J. Suarez Gonzalez, E. A. De Wolf, D. Di Croce, X. Janssen, J. Lauwers, M. Van De Klundert, H. Van Haevermaet, P. Van Mechelen, N. Van Remortel, A. Van Spilbeeck, S. Abu Zeid, F. Blekman, J. D’Hondt, I. De Bruyn, J. De Clercq, K. Deroover, G. Flouris, D. Lontkovskyi, S. Lowette, S. Moortgat, L. Moreels, A. Olbrechts, Q. Python, K. Skovpen, S. Tavernier, W. Van Doninck, P. Van Mulders, I. Van Parijs, H. Brun, B. Clerbaux, G. De Lentdecker, H. Delannoy, G. Fasanella, L. Favart, R. Goldouzian, A. Grebenyuk, G. Karapostoli, T. Lenzi, J. Luetic, T. Maerschalk, A. Marinov, A. Randle-conde, T. Seva, C. Vander Velde, P. Vanlaer, D. Vannerom, R. Yonamine, F. Zenoni, F. Zhang, A. Cimmino, T. Cornelis, D. Dobur, A. Fagot, M. Gul, I. Khvastunov, D. Poyraz, C. Roskas, S. Salva, M. Tytgat, W. Verbeke, N. Zaganidis, H. Bakhshiansohi, O. Bondu, S. Brochet, G. Bruno, A. Caudron, S. De Visscher, C. Delaere, M. Delcourt, B. Francois, A. Giammanco, A. Jafari, M. Komm, G. Krintiras, V. Lemaitre, A. Magitteri, A. Mertens, M. Musich, K. Piotrzkowski, L. Quertenmont, M. Vidal Marono, S. Wertz, N. Beliy, W. L. Aldá Júnior, F. L. Alves, G. A. Alves, L. Brito, M. Correa Martins Junior, C. Hensel, A. Moraes, M. E. Pol, P. Rebello Teles, E. Belchior Batista Das Chagas, W. Carvalho, J. Chinellato, A. Custódio, E. M. Da Costa, G. G. Da Silveira, D. De Jesus Damiao, S. Fonseca De Souza, L. M. Huertas Guativa, H. Malbouisson, M. Melo De Almeida, C. Mora Herrera, L. Mundim, H. Nogima, A. Santoro, A. Sznajder, E. J. Tonelli Manganote, F. Torres Da E Silva De Araujo, A. Vilela Pereira, S. Ahuja, C. A. Bernardes, T. R. Fernandez Perez Tomei, E. M. Gregores, P. G. Mercadante, C. S. Moon, S. F. Novaes, Sandra S. Padula, D. Romero Abad, J. C. Ruiz Vargas, A. Aleksandrov, R. Hadjiiska, P. Iaydjiev, M. Misheva, M. Rodozov, M. Shopova, S. Stoykova, G. Sultanov, A. Dimitrov, I. Glushkov, L. Litov, B. Pavlov, P. Petkov, W. Fang, X. Gao, M. Ahmad, J. G. Bian, G. M. Chen, H. S. Chen, M. Chen, Y. Chen, C. H. Jiang, D. Leggat, Z. Liu, F. Romeo, S. M. Shaheen, A. Spiezia, J. Tao, C. Wang, Z. Wang, E. Yazgan, H. Zhang, J. Zhao, Y. Ban, G. Chen, Q. Li, S. Liu, Y. Mao, S. J. Qian, D. Wang, Z. Xu, C. Avila, A. Cabrera, L. F. Chaparro Sierra, C. Florez, C. F. González Hernández, J. D. Ruiz Alvarez, B. Courbon, N. Godinovic, D. Lelas, I. Puljak, P. M. Ribeiro Cipriano, T. Sculac, Z. Antunovic, M. Kovac, V. Brigljevic, D. Ferencek, K. Kadija, B. Mesic, T. Susa, M. W. Ather, A. Attikis, G. Mavromanolakis, J. Mousa, C. Nicolaou, F. Ptochos, P. A. Razis, H. Rykaczewski, M. Finger, M. Finger, E. Carrera Jarrin, Y. Assran, S. Elgammal, A. Mahrous, R. K. Dewanjee, M. Kadastik, L. Perrini, M. Raidal, A. Tiko, C. Veelken, P. Eerola, J. Pekkanen, M. Voutilainen, J. Härkönen, T. Järvinen, V. Karimäki, R. Kinnunen, T. Lampén, K. Lassila-Perini, S. Lehti, T. Lindén, P. Luukka, E. Tuominen, J. Tuominiemi, E. Tuovinen, J. Talvitie, T. Tuuva, M. Besancon, F. Couderc, M. Dejardin, D. Denegri, J. L. Faure, F. Ferri, S. Ganjour, S. Ghosh, A. Givernaud, P. Gras, G. Hamel de Monchenault, P. Jarry, I. Kucher, E. Locci, M. Machet, J. Malcles, G. Negro, J. Rander, A. Rosowsky, M. Ö. Sahin, M. Titov, A. Abdulsalam, I. Antropov, S. Baffioni, F. Beaudette, P. Busson, L. Cadamuro, C. Charlot, O. Davignon, R. Granier de Cassagnac, M. Jo, S. Lisniak, A. Lobanov, J. Martin Blanco, M. Nguyen, C. Ochando, G. Ortona, P. Paganini, P. Pigard, S. Regnard, R. Salerno, J. B. Sauvan, Y. Sirois, A. G. Stahl Leiton, T. Strebler, Y. Yilmaz, A. Zabi, J.-L. Agram, J. Andrea, D. Bloch, J.-M. Brom, M. Buttignol, E. C. Chabert, N. Chanon, C. Collard, E. Conte, X. Coubez, J.-C. Fontaine, D. Gelé, U. Goerlach, M. Jansová, A.-C. Le Bihan, N. Tonon, P. Van Hove, S. Gadrat, S. Beauceron, C. Bernet, G. Boudoul, R. Chierici, D. Contardo, P. Depasse, H. El Mamouni, J. Fay, L. Finco, S. Gascon, M. Gouzevitch, G. Grenier, B. Ille, F. Lagarde, I. B. Laktineh, M. Lethuillier, L. Mirabito, A. L. Pequegnot, S. Perries, A. Popov, V. Sordini, M. Vander Donckt, S. Viret, A. Khvedelidze, Z. Tsamalaidze, C. Autermann, S. Beranek, L. Feld, M. K. Kiesel, K. Klein, M. Lipinski, M. Preuten, C. Schomakers, J. Schulz, T. Verlage, A. Albert, M. Brodski, E. Dietz-Laursonn, D. Duchardt, M. Endres, M. Erdmann, S. Erdweg, T. Esch, R. Fischer, A. Güth, M. Hamer, T. Hebbeker, C. Heidemann, K. Hoepfner, S. Knutzen, M. Merschmeyer, A. Meyer, P. Millet, S. Mukherjee, M. Olschewski, K. Padeken, T. Pook, M. Radziej, H. Reithler, M. Rieger, F. Scheuch, D. Teyssier, S. Thüer, G. Flügge, B. Kargoll, T. Kress, A. Künsken, J. Lingemann, T. Müller, A. Nehrkorn, A. Nowack, C. Pistone, O. Pooth, A. Stahl, M. Aldaya Martin, T. Arndt, C. Asawatangtrakuldee, K. Beernaert, O. Behnke, U. Behrens, A. A. Bin Anuar, K. Borras, V. Botta, A. Campbell, P. Connor, C. Contreras-Campana, F. Costanza, C. Diez Pardos, G. Eckerlin, D. Eckstein, T. Eichhorn, E. Eren, E. Gallo, J. Garay Garcia, A. Geiser, A. Gizhko, J. M. Grados Luyando, A. Grohsjean, P. Gunnellini, A. Harb, J. Hauk, M. Hempel, H. Jung, A. Kalogeropoulos, M. Kasemann, J. Keaveney, C. Kleinwort, I. Korol, D. Krücker, W. Lange, A. Lelek, T. Lenz, J. Leonard, K. Lipka, W. Lohmann, R. Mankel, I.-A. Melzer-Pellmann, A. B. Meyer, G. Mittag, J. Mnich, A. Mussgiller, E. Ntomari, D. Pitzl, R. Placakyte, A. Raspereza, B. Roland, M. Savitskyi, P. Saxena, R. Shevchenko, S. Spannagel, N. Stefaniuk, G. P. Van Onsem, R. Walsh, Y. Wen, K. Wichmann, C. Wissing, O. Zenaiev, S. Bein, V. Blobel, M. Centis Vignali, A. R. Draeger, T. Dreyer, E. Garutti, D. Gonzalez, J. Haller, M. Hoffmann, A. Junkes, A. Karavdina, R. Klanner, R. Kogler, N. Kovalchuk, S. Kurz, T. Lapsien, I. Marchesini, D. Marconi, M. Meyer, M. Niedziela, D. Nowatschin, F. Pantaleo, T. Peiffer, A. Perieanu, C. Scharf, P. Schleper, A. Schmidt, S. Schumann, J. Schwandt, J. Sonneveld, H. Stadie, G. Steinbrück, F. M. Stober, M. Stöver, H. Tholen, D. Troendle, E. Usai, L. Vanelderen, A. Vanhoefer, B. Vormwald, M. Akbiyik, C. Barth, S. Baur, E. Butz, R. Caspart, T. Chwalek, F. Colombo, W. De Boer, A. Dierlamm, B. Freund, R. Friese, M. Giffels, A. Gilbert, D. Haitz, F. Hartmann, S. M. Heindl, U. Husemann, F. Kassel, S. Kudella, H. Mildner, M. U. Mozer, Th. Müller, M. Plagge, G. Quast, K. Rabbertz, M. Schröder, I. Shvetsov, G. Sieber, H. J. Simonis, R. Ulrich, S. Wayand, M. Weber, T. Weiler, S. Williamson, C. Wöhrmann, R. Wolf, G. Anagnostou, G. Daskalakis, T. Geralis, V. A. Giakoumopoulou, A. Kyriakis, D. Loukas, I. Topsis-Giotis, S. Kesisoglou, A. Panagiotou, N. Saoulidou, I. Evangelou, C. Foudas, P. Kokkas, N. Manthos, I. Papadopoulos, E. Paradas, J. Strologas, F. A. Triantis, M. Csanad, N. Filipovic, G. Pasztor, G. Bencze, C. Hajdu, D. Horvath, Hunyadi, F. Sikler, V. Veszpremi, G. Vesztergombi, A. J. Zsigmond, N. Beni, S. Czellar, J. Karancsi, A. Makovec, J. Molnar, Z. Szillasi, M. Bartók, P. Raics, Z. L. Trocsanyi, B. Ujvari, S. Choudhury, J. R. Komaragiri, S. Bahinipati, S. Bhowmik, P. Mal, K. Mandal, A. Nayak, D. K. Sahoo, N. Sahoo, S. K. Swain, S. Bansal, S. B. Beri, V. Bhatnagar, R. Chawla, N. Dhingra, U. Bhawandeep, A. K. Kalsi, A. Kaur, M. Kaur, R. Kumar, P. Kumari, A. Mehta, J. B. Singh, G. Walia, Ashok Kumar, Aashaq Shah, A. Bhardwaj, S. Chauhan, B. C. Choudhary, R. B. Garg, S. Keshri, A. Kumar, S. Malhotra, M. Naimuddin, K. Ranjan, R. Sharma, V. Sharma, R. Bhardwaj, R. Bhattacharya, S. Bhattacharya, S. Dey, S. Dutt, S. Dutta, S. Ghosh, N. Majumdar, A. Modak, K. Mondal, S. Mukhopadhyay, S. Nandan, A. Purohit, A. Roy, D. Roy, S. Roy Chowdhury, S. Sarkar, M. Sharan, S. Thakur, P. K. Behera, R. Chudasama, D. Dutta, V. Jha, V. Kumar, A. K. Mohanty, P. K. Netrakanti, L. M. Pant, P. Shukla, A. Topkar, T. Aziz, S. Dugad, B. Mahakud, S. Mitra, G. B. Mohanty, B. Parida, N. Sur, B. Sutar, S. Banerjee, S. Bhattacharya, S. Chatterjee, P. Das, M. Guchait, Sa. Jain, S. Kumar, M. Maity, G. Majumder, K. Mazumdar, T. Sarkar, N. Wickramage, S. Chauhan, S. Dube, V. Hegde, A. Kapoor, K. Kothekar, S. Pandey, A. Rane, S. Sharma, S. Chenarani, E. Eskandari Tadavani, S. M. Etesami, M. Khakzad, M. Mohammadi Najafabadi, M. Naseri, S. Paktinat Mehdiabadi, F. Rezaei Hosseinabadi, B. Safarzadeh, M. Zeinali, M. Felcini, M. Grunewald, M. Abbrescia, C. Calabria, C. Caputo, A. Colaleo, D. Creanza, L. Cristella, N. De Filippis, M. De Palma, F. Errico, L. Fiore, G. Iaselli, G. Maggi, M. Maggi, G. Miniello, S. My, S. Nuzzo, A. Pompili, G. Pugliese, R. Radogna, A. Ranieri, G. Selvaggi, A. Sharma, L. Silvestris, R. Venditti, P. Verwilligen, G. Abbiendi, C. Battilana, D. Bonacorsi, S. Braibant-Giacomelli, L. Brigliadori, R. Campanini, P. Capiluppi, A. Castro, F. R. Cavallo, S. S. Chhibra, G. Codispoti, M. Cuffiani, G. M. Dallavalle, F. Fabbri, A. Fanfani, D. Fasanella, P. Giacomelli, L. Guiducci, S. Marcellini, G. Masetti, F. L. Navarria, A. Perrotta, A. M. Rossi, T. Rovelli, G. P. Siroli, N. Tosi, S. Albergo, S. Costa, A. Di Mattia, F. Giordano, R. Potenza, A. Tricomi, C. Tuve, G. Barbagli, K. Chatterjee, V. Ciulli, C. Civinini, R. D’Alessandro, E. Focardi, P. Lenzi, M. Meschini, S. Paoletti, L. Russo, G. Sguazzoni, D. Strom, L. Viliani, L. Benussi, S. Bianco, F. Fabbri, D. Piccolo, F. Primavera, V. Calvelli, F. Ferro, E. Robutti, S. Tosi, L. Brianza, F. Brivio, V. Ciriolo, M. E. Dinardo, S. Fiorendi, S. Gennai, A. Ghezzi, P. Govoni, M. Malberti, S. Malvezzi, R. A. Manzoni, D. Menasce, L. Moroni, M. Paganoni, K. Pauwels, D. Pedrini, S. Pigazzini, S. Ragazzi, T. Tabarelli de Fatis, S. Buontempo, N. Cavallo, S. Di Guida, M. Esposito, F. Fabozzi, F. Fienga, A. O. M. Iorio, W. A. Khan, G. Lanza, L. Lista, S. Meola, P. Paolucci, C. Sciacca, F. Thyssen, P. Azzi, N. Bacchetta, L. Benato, D. Bisello, A. Boletti, R. Carlin, A. Carvalho Antunes De Oliveira, P. Checchia, P. De Castro Manzano, T. Dorigo, U. Dosselli, F. Gasparini, U. Gasparini, A. Gozzelino, S. Lacaprara, M. Margoni, A. T. Meneguzzo, N. Pozzobon, P. Ronchese, R. Rossin, F. Simonetto, E. Torassa, M. Zanetti, P. Zotto, G. Zumerle, A. Braghieri, F. Fallavollita, A. Magnani, P. Montagna, S. P. Ratti, V. Re, M. Ressegotti, C. Riccardi, P. Salvini, I. Vai, P. Vitulo, L. Alunni Solestizi, G. M. Bilei, D. Ciangottini, L. Fanò, P. Lariccia, R. Leonardi, G. Mantovani, V. Mariani, M. Menichelli, A. Saha, A. Santocchia, D. Spiga, K. Androsov, P. Azzurri, G. Bagliesi, J. Bernardini, T. Boccali, L. Borrello, R. Castaldi, M. A. Ciocci, R. Dell’Orso, G. Fedi, L. Giannini, A. Giassi, M. T. Grippo, F. Ligabue, T. Lomtadze, E. Manca, G. Mandorli, L. Martini, A. Messineo, F. Palla, A. Rizzi, A. Savoy-Navarro, P. Spagnolo, R. Tenchini, G. Tonelli, A. Venturi, P. G. Verdini, L. Barone, F. Cavallari, M. Cipriani, D. Del Re, M. Diemoz, S. Gelli, E. Longo, F. Margaroli, B. Marzocchi, P. Meridiani, G. Organtini, R. Paramatti, F. Preiato, S. Rahatlou, C. Rovelli, F. Santanastasio, N. Amapane, R. Arcidiacono, S. Argiro, M. Arneodo, N. Bartosik, R. Bellan, C. Biino, N. Cartiglia, F. Cenna, M. Costa, R. Covarelli, A. Degano, N. Demaria, B. Kiani, C. Mariotti, S. Maselli, E. Migliore, V. Monaco, E. Monteil, M. Monteno, M. M. Obertino, L. Pacher, N. Pastrone, M. Pelliccioni, G. L. Pinna Angioni, F. Ravera, A. Romero, M. Ruspa, R. Sacchi, K. Shchelina, V. Sola, A. Solano, A. Staiano, P. Traczyk, S. Belforte, M. Casarsa, F. Cossutti, G. Della Ricca, A. Zanetti, D. H. Kim, G. N. Kim, M. S. Kim, J. Lee, S. Lee, S. W. Lee, Y. D. Oh, S. Sekmen, D. C. Son, Y. C. Yang, A. Lee, H. Kim, D. H. Moon, G. Oh, J. A. Brochero Cifuentes, J. Goh, T. J. Kim, S. Cho, S. Choi, Y. Go, D. Gyun, S. Ha, B. Hong, Y. Jo, Y. Kim, K. Lee, K. S. Lee, S. Lee, J. Lim, S. K. Park, Y. Roh, J. Almond, J. Kim, J. S. Kim, H. Lee, K. Lee, K. Nam, S. B. Oh, B. C. Radburn-Smith, S. h. Seo, U. K. Yang, H. D. Yoo, G. B. Yu, M. Choi, H. Kim, J. H. Kim, J. S. H. Lee, I. C. Park, G. Ryu, Y. Choi, C. Hwang, J. Lee, I. Yu, V. Dudenas, A. Juodagalvis, J. Vaitkus, I. Ahmed, Z. A. Ibrahim, M. A. B. Md Ali, F. Mohamad Idris, W. A. T. Wan Abdullah, M. N. Yusli, Z. Zolkapli, H. Castilla-Valdez, E. De La Cruz-Burelo, I. Heredia-De La Cruz, R. Lopez-Fernandez, J. Mejia Guisao, A. Sanchez-Hernandez, S. Carrillo Moreno, C. Oropeza Barrera, F. Vazquez Valencia, I. Pedraza, H. A. Salazar Ibarguen, C. Uribe Estrada, A. Morelos Pineda, D. Krofcheck, P. H. Butler, A. Ahmad, M. Ahmad, Q. Hassan, H. R. Hoorani, A. Saddique, M. A. Shah, M. Shoaib, M. Waqas, H. Bialkowska, M. Bluj, B. Boimska, T. Frueboes, M. Górski, M. Kazana, K. Nawrocki, K. Romanowska-Rybinska, M. Szleper, P. Zalewski, K. Bunkowski, A. Byszuk, K. Doroba, A. Kalinowski, M. Konecki, J. Krolikowski, M. Misiura, M. Olszewski, A. Pyskir, M. Walczak, P. Bargassa, C. Beirão Da Cruz E Silva, B. Calpas, A. Di Francesco, P. Faccioli, M. Gallinaro, J. Hollar, N. Leonardo, L. Lloret Iglesias, M. V. Nemallapudi, J. Seixas, O. Toldaiev, D. Vadruccio, J. Varela, S. Afanasiev, P. Bunin, M. Gavrilenko, I. Golutvin, I. Gorbunov, A. Kamenev, V. Karjavin, A. Lanev, A. Malakhov, V. Matveev, V. Palichik, V. Perelygin, S. Shmatov, S. Shulha, N. Skatchkov, V. Smirnov, N. Voytishin, A. Zarubin, Y. Ivanov, V. Kim, E. Kuznetsova, P. Levchenko, V. Murzin, V. Oreshkin, I. Smirnov, V. Sulimov, L. Uvarov, S. Vavilov, A. Vorobyev, Yu. Andreev, A. Dermenev, S. Gninenko, N. Golubev, A. Karneyeu, M. Kirsanov, N. Krasnikov, A. Pashenkov, D. Tlisov, A. Toropin, V. Epshteyn, V. Gavrilov, N. Lychkovskaya, V. Popov, I. Pozdnyakov, G. Safronov, A. Spiridonov, A. Stepennov, M. Toms, E. Vlasov, A. Zhokin, T. Aushev, A. Bylinkin, R. Chistov, M. Danilov, P. Parygin, D. Philippov, S. Polikarpov, E. Tarkovskii, V. Andreev, M. Azarkin, I. Dremin, M. Kirakosyan, A. Terkulov, A. Baskakov, A. Belyaev, E. Boos, M. Dubinin, L. Dudko, A. Ershov, A. Gribushin, V. Klyukhin, O. Kodolova, I. Lokhtin, I. Miagkov, S. Obraztsov, S. Petrushanko, V. Savrin, A. Snigirev, V. Blinov, Y. Skovpen, D. Shtol, I. Azhgirey, I. Bayshev, S. Bitioukov, D. Elumakhov, V. Kachanov, A. Kalinin, D. Konstantinov, V. Krychkine, V. Petrov, R. Ryutin, A. Sobol, S. Troshin, N. Tyurin, A. Uzunian, A. Volkov, P. Adzic, P. Cirkovic, D. Devetak, M. Dordevic, J. Milosevic, V. Rekovic, J. Alcaraz Maestre, M. Barrio Luna, M. Cerrada, N. Colino, B. De La Cruz, A. Delgado Peris, A. Escalante Del Valle, C. Fernandez Bedoya, J. P. Fernández Ramos, J. Flix, M. C. Fouz, P. Garcia-Abia, O. Gonzalez Lopez, S. Goy Lopez, J. M. Hernandez, M. I. Josa, A. Pérez-Calero Yzquierdo, J. Puerta Pelayo, A. Quintario Olmeda, I. Redondo, L. Romero, M. S. Soares, A. lvarez Fernández, J. F. de Trocóniz, M. Missiroli, D. Moran, J. Cuevas, C. Erice, J. Fernandez Menendez, I. Gonzalez Caballero, J. R. González Fernández, E. Palencia Cortezon, S. Sanchez Cruz, I. Suárez Andrés, P. Vischia, J. M. Vizan Garcia, I. J. Cabrillo, A. Calderon, B. Chazin Quero, E. Curras, M. Fernandez, J. Garcia-Ferrero, G. Gomez, A. Lopez Virto, J. Marco, C. Martinez Rivero, P. Martinez Ruiz del Arbol, F. Matorras, J. Piedra Gomez, T. Rodrigo, A. Ruiz-Jimeno, L. Scodellaro, N. Trevisani, I. Vila, R. Vilar Cortabitarte, D. Abbaneo, E. Auffray, P. Baillon, A. H. Ball, D. Barney, M. Bianco, P. Bloch, A. Bocci, C. Botta, T. Camporesi, R. Castello, M. Cepeda, G. Cerminara, E. Chapon, Y. Chen, D. d’Enterria, A. Dabrowski, V. Daponte, A. David, M. De Gruttola, A. De Roeck, E. Di Marco, M. Dobson, B. Dorney, T. du Pree, M. Dünser, N. Dupont, A. Elliott-Peisert, P. Everaerts, G. Franzoni, J. Fulcher, W. Funk, D. Gigi, K. Gill, F. Glege, D. Gulhan, S. Gundacker, M. Guthoff, P. Harris, J. Hegeman, V. Innocente, P. Janot, O. Karacheban, J. Kieseler, H. Kirschenmann, V. Knünz, A. Kornmayer, M. J. Kortelainen, C. Lange, P. Lecoq, C. Lourenço, M. T. Lucchini, L. Malgeri, M. Mannelli, A. Martelli, F. Meijers, J. A. Merlin, S. Mersi, E. Meschi, P. Milenovic, F. Moortgat, M. Mulders, H. Neugebauer, S. Orfanelli, L. Orsini, L. Pape, E. Perez, M. Peruzzi, A. Petrilli, G. Petrucciani, A. Pfeiffer, M. Pierini, A. Racz, T. Reis, G. Rolandi, M. Rovere, H. Sakulin, C. Schäfer, C. Schwick, M. Seidel, M. Selvaggi, A. Sharma, P. Silva, P. Sphicas, J. Steggemann, M. Stoye, M. Tosi, D. Treille, A. Triossi, A. Tsirou, V. Veckalns, G. I. Veres, M. Verweij, N. Wardle, W. D. Zeuner, W. Bertl, K. Deiters, W. Erdmann, R. Horisberger, Q. Ingram, H. C. Kaestli, D. Kotlinski, U. Langenegger, T. Rohe, S. A. Wiederkehr, F. Bachmair, L. Bäni, P. Berger, L. Bianchini, B. Casal, G. Dissertori, M. Dittmar, M. Donegà, C. Grab, C. Heidegger, D. Hits, J. Hoss, G. Kasieczka, T. Klijnsma, W. Lustermann, B. Mangano, M. Marionneau, M. T. Meinhard, D. Meister, F. Micheli, P. Musella, F. Nessi-Tedaldi, F. Pandolfi, J. Pata, F. Pauss, G. Perrin, L. Perrozzi, M. Quittnat, M. Rossini, M. Schönenberger, L. Shchutska, A. Starodumov, V. R. Tavolaro, K. Theofilatos, M. L. Vesterbacka Olsson, R. Wallny, A. Zagozdzinska, D. H. Zhu, T. K. Aarrestad, C. Amsler, L. Caminada, M. F. Canelli, A. De Cosa, S. Donato, C. Galloni, A. Hinzmann, T. Hreus, B. Kilminster, J. Ngadiuba, D. Pinna, G. Rauco, P. Robmann, D. Salerno, C. Seitz, A. Zucchetta, V. Candelise, T. H. Doan, Sh. Jain, R. Khurana, M. Konyushikhin, C. M. Kuo, W. Lin, A. Pozdnyakov, S. S. Yu, Arun Kumar, P. Chang, Y. Chao, K. F. Chen, P. H. Chen, F. Fiori, W.-S. Hou, Y. Hsiung, Y. F. Liu, R.-S. Lu, M. Miñano Moya, E. Paganis, A. Psallidas, J. f. Tsai, B. Asavapibhop, K. Kovitanggoon, G. Singh, N. Srimanobhas, A. Adiguzel, F. Boran, S. Cerci, S. Damarseckin, Z. S. Demiroglu, C. Dozen, I. Dumanoglu, S. Girgis, G. Gokbulut, Y. Guler, I. Hos, E. E. Kangal, O. Kara, U. Kiminsu, M. Oglakci, G. Onengut, K. Ozdemir, D. Sunar Cerci, B. Tali, H. Topakli, S. Turkcapar, I. S. Zorbakir, C. Zorbilmez, B. Bilin, G. Karapinar, K. Ocalan, M. Yalvac, M. Zeyrek, E. Gülmez, M. Kaya, O. Kaya, S. Tekten, E. A. Yetkin, M. N. Agaras, S. Atay, A. Cakir, K. Cankocak, B. Grynyov, L. Levchuk, P. Sorokin, R. Aggleton, F. Ball, L. Beck, J. J. Brooke, D. Burns, E. Clement, D. Cussans, H. Flacher, J. Goldstein, M. Grimes, G. P. Heath, H. F. Heath, J. Jacob, L. Kreczko, C. Lucas, D. M. Newbold, S. Paramesvaran, A. Poll, T. Sakuma, S. Seif El Nasr-storey, D. Smith, V. J. Smith, K. W. Bell, A. Belyaev, C. Brew, R. M. Brown, L. Calligaris, D. Cieri, D. J. A. Cockerill, J. A. Coughlan, K. Harder, S. Harper, E. Olaiya, D. Petyt, C. H. Shepherd-Themistocleous, A. Thea, I. R. Tomalin, T. Williams, M. Baber, R. Bainbridge, S. Breeze, O. Buchmuller, A. Bundock, S. Casasso, M. Citron, D. Colling, L. Corpe, P. Dauncey, G. Davies, A. De Wit, M. Della Negra, R. Di Maria, P. Dunne, A. Elwood, D. Futyan, Y. Haddad, G. Hall, G. Iles, T. James, R. Lane, C. Laner, L. Lyons, A.-M. Magnan, S. Malik, L. Mastrolorenzo, T. Matsushita, J. Nash, A. Nikitenko, J. Pela, M. Pesaresi, D. M. Raymond, A. Richards, A. Rose, E. Scott, C. Seez, A. Shtipliyski, S. Summers, A. Tapper, K. Uchida, M. Vazquez Acosta, T. Virdee, D. Winterbottom, J. Wright, S. C. Zenz, J. E. Cole, P. R. Hobson, A. Khan, P. Kyberd, I. D. Reid, P. Symonds, L. Teodorescu, M. Turner, A. Borzou, K. Call, J. Dittmann, K. Hatakeyama, H. Liu, N. Pastika, R. Bartek, A. Dominguez, A. Buccilli, S. I. Cooper, C. Henderson, P. Rumerio, C. West, D. Arcaro, A. Avetisyan, T. Bose, D. Gastler, D. Rankin, C. Richardson, J. Rohlf, L. Sulak, D. Zou, G. Benelli, D. Cutts, A. Garabedian, J. Hakala, U. Heintz, J. M. Hogan, K. H. M. Kwok, E. Laird, G. Landsberg, Z. Mao, M. Narain, S. Piperov, S. Sagir, R. Syarif, D. Yu, R. Band, C. Brainerd, D. Burns, M. Calderon De La Barca Sanchez, M. Chertok, J. Conway, R. Conway, P. T. Cox, R. Erbacher, C. Flores, G. Funk, M. Gardner, W. Ko, R. Lander, C. Mclean, M. Mulhearn, D. Pellett, J. Pilot, S. Shalhout, M. Shi, J. Smith, M. Squires, D. Stolp, K. Tos, M. Tripathi, Z. Wang, M. Bachtis, C. Bravo, R. Cousins, A. Dasgupta, A. Florent, J. Hauser, M. Ignatenko, N. Mccoll, D. Saltzberg, C. Schnaible, V. Valuev, E. Bouvier, K. Burt, R. Clare, J. Ellison, J. W. Gary, S. M. A. Ghiasi Shirazi, G. Hanson, J. Heilman, P. Jandir, E. Kennedy, F. Lacroix, O. R. Long, M. Olmedo Negrete, M. I. Paneva, A. Shrinivas, W. Si, H. Wei, S. Wimpenny, B. R. Yates, J. G. Branson, S. Cittolin, M. Derdzinski, B. Hashemi, A. Holzner, D. Klein, G. Kole, V. Krutelyov, J. Letts, I. Macneill, M. Masciovecchio, D. Olivito, S. Padhi, M. Pieri, M. Sani, V. Sharma, S. Simon, M. Tadel, A. Vartak, S. Wasserbaech, J. Wood, F. Würthwein, A. Yagil, G. Zevi Della Porta, N. Amin, R. Bhandari, J. Bradmiller-Feld, C. Campagnari, A. Dishaw, V. Dutta, M. Franco Sevilla, C. George, F. Golf, L. Gouskos, J. Gran, R. Heller, J. Incandela, S. D. Mullin, A. Ovcharova, H. Qu, J. Richman, D. Stuart, I. Suarez, J. Yoo, D. Anderson, J. Bendavid, A. Bornheim, J. M. Lawhorn, H. B. Newman, T. Nguyen, C. Pena, M. Spiropulu, J. R. Vlimant, S. Xie, Z. Zhang, R. Y. Zhu, M. B. Andrews, T. Ferguson, T. Mudholkar, M. Paulini, J. Russ, M. Sun, H. Vogel, I. Vorobiev, M. Weinberg, J. P. Cumalat, W. T. Ford, F. Jensen, A. Johnson, M. Krohn, S. Leontsinis, T. Mulholland, K. Stenson, S. R. Wagner, J. Alexander, J. Chaves, J. Chu, S. Dittmer, K. Mcdermott, N. Mirman, J. R. Patterson, A. Rinkevicius, A. Ryd, L. Skinnari, L. Soffi, S. M. Tan, Z. Tao, J. Thom, J. Tucker, P. Wittich, M. Zientek, S. Abdullin, M. Albrow, G. Apollinari, A. Apresyan, A. Apyan, S. Banerjee, L. A. T. Bauerdick, A. Beretvas, J. Berryhill, P. C. Bhat, G. Bolla, K. Burkett, J. N. Butler, A. Canepa, G. B. Cerati, H. W. K. Cheung, F. Chlebana, M. Cremonesi, J. Duarte, V. D. Elvira, J. Freeman, Z. Gecse, E. Gottschalk, L. Gray, D. Green, S. Grünendahl, O. Gutsche, R. M. Harris, S. Hasegawa, J. Hirschauer, Z. Hu, B. Jayatilaka, S. Jindariani, M. Johnson, U. Joshi, B. Klima, B. Kreis, S. Lammel, D. Lincoln, R. Lipton, M. Liu, T. Liu, R. Lopes De Sá, J. Lykken, K. Maeshima, N. Magini, J. M. Marraffino, S. Maruyama, D. Mason, P. McBride, P. Merkel, S. Mrenna, S. Nahn, V. O’Dell, K. Pedro, O. Prokofyev, G. Rakness, L. Ristori, B. Schneider, E. Sexton-Kennedy, A. Soha, W. J. Spalding, L. Spiegel, S. Stoynev, J. Strait, N. Strobbe, L. Taylor, S. Tkaczyk, N. V. Tran, L. Uplegger, E. W. Vaandering, C. Vernieri, M. Verzocchi, R. Vidal, M. Wang, H. A. Weber, A. Whitbeck, D. Acosta, P. Avery, P. Bortignon, A. Brinkerhoff, A. Carnes, M. Carver, D. Curry, S. Das, R. D. Field, I. K. Furic, J. Konigsberg, A. Korytov, K. Kotov, P. Ma, K. Matchev, H. Mei, G. Mitselmakher, D. Rank, D. Sperka, N. Terentyev, L. Thomas, J. Wang, S. Wang, J. Yelton, Y. R. Joshi, S. Linn, P. Markowitz, G. Martinez, J. L. Rodriguez, A. Ackert, T. Adams, A. Askew, S. Hagopian, V. Hagopian, K. F. Johnson, T. Kolberg, T. Perry, H. Prosper, A. Santra, R. Yohay, M. M. Baarmand, V. Bhopatkar, S. Colafranceschi, M. Hohlmann, D. Noonan, T. Roy, F. Yumiceva, M. R. Adams, L. Apanasevich, D. Berry, R. R. Betts, R. Cavanaugh, X. Chen, O. Evdokimov, C. E. Gerber, D. A. Hangal, D. J. Hofman, K. Jung, J. Kamin, I. D. Sandoval Gonzalez, M. B. Tonjes, H. Trauger, N. Varelas, H. Wang, Z. Wu, J. Zhang, B. Bilki, W. Clarida, K. Dilsiz, S. Durgut, R. P. Gandrajula, M. Haytmyradov, V. Khristenko, J.-P. Merlo, H. Mermerkaya, A. Mestvirishvili, A. Moeller, J. Nachtman, H. Ogul, Y. Onel, F. Ozok, A. Penzo, C. Snyder, E. Tiras, J. Wetzel, K. Yi, B. Blumenfeld, A. Cocoros, N. Eminizer, D. Fehling, L. Feng, A. V. Gritsan, P. Maksimovic, J. Roskes, U. Sarica, M. Swartz, M. Xiao, C. You, A. Al-bataineh, P. Baringer, A. Bean, S. Boren, J. Bowen, J. Castle, S. Khalil, A. Kropivnitskaya, D. Majumder, W. Mcbrayer, M. Murray, C. Royon, S. Sanders, E. Schmitz, R. Stringer, J. D. Tapia Takaki, Q. Wang, A. Ivanov, K. Kaadze, Y. Maravin, A. Mohammadi, L. K. Saini, N. Skhirtladze, S. Toda, F. Rebassoo, D. Wright, C. Anelli, A. Baden, O. Baron, A. Belloni, B. Calvert, S. C. Eno, C. Ferraioli, N. J. Hadley, S. Jabeen, G. Y. Jeng, R. G. Kellogg, J. Kunkle, A. C. Mignerey, F. Ricci-Tam, Y. H. Shin, A. Skuja, S. C. Tonwar, D. Abercrombie, B. Allen, V. Azzolini, R. Barbieri, A. Baty, R. Bi, S. Brandt, W. Busza, I. A. Cali, M. D’Alfonso, Z. Demiragli, G. Gomez Ceballos, M. Goncharov, D. Hsu, Y. Iiyama, G. M. Innocenti, M. Klute, D. Kovalskyi, Y. S. Lai, Y.-J. Lee, A. Levin, P. D. Luckey, B. Maier, A. C. Marini, C. Mcginn, C. Mironov, S. Narayanan, X. Niu, C. Paus, C. Roland, G. Roland, J. Salfeld-Nebgen, G. S. F. Stephans, K. Tatar, D. Velicanu, J. Wang, T. W. Wang, B. Wyslouch, A. C. Benvenuti, R. M. Chatterjee, A. Evans, P. Hansen, S. Kalafut, Y. Kubota, Z. Lesko, J. Mans, S. Nourbakhsh, N. Ruckstuhl, R. Rusack, J. Turkewitz, J. G. Acosta, S. Oliveros, E. Avdeeva, K. Bloom, D. R. Claes, C. Fangmeier, R. Gonzalez Suarez, R. Kamalieddin, I. Kravchenko, J. Monroy, J. E. Siado, G. R. Snow, B. Stieger, M. Alyari, J. Dolen, A. Godshalk, C. Harrington, I. Iashvili, D. Nguyen, A. Parker, S. Rappoccio, B. Roozbahani, G. Alverson, E. Barberis, A. Hortiangtham, A. Massironi, D. M. Morse, D. Nash, T. Orimoto, R. Teixeira De Lima, D. Trocino, R.-J. Wang, D. Wood, S. Bhattacharya, O. Charaf, K. A. Hahn, N. Mucia, N. Odell, B. Pollack, M. H. Schmitt, K. Sung, M. Trovato, M. Velasco, N. Dev, M. Hildreth, K. Hurtado Anampa, C. Jessop, D. J. Karmgard, N. Kellams, K. Lannon, N. Loukas, N. Marinelli, F. Meng, C. Mueller, Y. Musienko, M. Planer, A. Reinsvold, R. Ruchti, G. Smith, S. Taroni, M. Wayne, M. Wolf, A. Woodard, J. Alimena, L. Antonelli, B. Bylsma, L. S. Durkin, S. Flowers, B. Francis, A. Hart, C. Hill, W. Ji, B. Liu, W. Luo, D. Puigh, B. L. Winer, H. W. Wulsin, A. Benaglia, S. Cooperstein, O. Driga, P. Elmer, J. Hardenbrook, P. Hebda, D. Lange, J. Luo, D. Marlow, K. Mei, I. Ojalvo, J. Olsen, C. Palmer, P. Piroué, D. Stickland, A. Svyatkovskiy, C. Tully, S. Malik, S. Norberg, A. Barker, V. E. Barnes, S. Folgueras, L. Gutay, M. K. Jha, M. Jones, A. W. Jung, A. Khatiwada, D. H. Miller, N. Neumeister, J. F. Schulte, J. Sun, F. Wang, W. Xie, T. Cheng, N. Parashar, J. Stupak, A. Adair, B. Akgun, Z. Chen, K. M. Ecklund, F. J. M. Geurts, M. Guilbaud, W. Li, B. Michlin, M. Northup, B. P. Padley, J. Roberts, J. Rorie, Z. Tu, J. Zabel, A. Bodek, P. de Barbaro, R. Demina, Y. t. Duh, T. Ferbel, M. Galanti, A. Garcia-Bellido, J. Han, O. Hindrichs, A. Khukhunaishvili, K. H. Lo, P. Tan, M. Verzetti, R. Ciesielski, K. Goulianos, C. Mesropian, A. Agapitos, J. P. Chou, Y. Gershtein, T. A. Gómez Espinosa, E. Halkiadakis, M. Heindl, E. Hughes, S. Kaplan, R. Kunnawalkam Elayavalli, S. Kyriacou, A. Lath, R. Montalvo, K. Nash, M. Osherson, H. Saka, S. Salur, S. Schnetzer, D. Sheffield, S. Somalwar, R. Stone, S. Thomas, P. Thomassen, M. Walker, M. Foerster, J. Heideman, G. Riley, K. Rose, S. Spanier, K. Thapa, O. Bouhali, A. Castaneda Hernandez, A. Celik, M. Dalchenko, M. De Mattia, A. Delgado, S. Dildick, R. Eusebi, J. Gilmore, T. Huang, T. Kamon, R. Mueller, Y. Pakhotin, R. Patel, A. Perloff, L. Perniè, D. Rathjens, A. Safonov, A. Tatarinov, K. A. Ulmer, N. Akchurin, J. Damgov, F. De Guio, P. R. Dudero, J. Faulkner, E. Gurpinar, S. Kunori, K. Lamichhane, S. W. Lee, T. Libeiro, T. Peltola, S. Undleeb, I. Volobouev, Z. Wang, S. Greene, A. Gurrola, R. Janjam, W. Johns, C. Maguire, A. Melo, H. Ni, P. Sheldon, S. Tuo, J. Velkovska, Q. Xu, M. W. Arenton, P. Barria, B. Cox, R. Hirosky, A. Ledovskoy, H. Li, C. Neu, T. Sinthuprasith, X. Sun, Y. Wang, E. Wolfe, F. Xia, C. Clarke, R. Harr, P. E. Karchin, J. Sturdy, S. Zaleski, J. Buchanan, C. Caillol, S. Dasu, L. Dodd, S. Duric, B. Gomber, M. Grothe, M. Herndon, A. Hervé, U. Hussain, P. Klabbers, A. Lanaro, A. Levine, K. Long, R. Loveless, G. A. Pierro, G. Polese, T. Ruggles, A. Savin, N. Smith, W. H. Smith, D. Taylor, N. Woods, CMS Collaboration

**Affiliations:** 1 Yerevan Physics Institute, Yerevan, Armenia; 2 Institut für Hochenergiephysik, Wien, Austria; 3 Institute for Nuclear Problems, Minsk, Belarus; 4 Universiteit Antwerpen, Antwerpen, Belgium; 5 Vrije Universiteit Brussel, Brussel, Belgium; 6 Université Libre de Bruxelles, Bruxelles, Belgium; 7 Ghent University, Ghent, Belgium; 8 Université Catholique de Louvain, Louvain-la-Neuve, Belgium; 9 Université de Mons, Mons, Belgium; 10 Centro Brasileiro de Pesquisas Fisicas, Rio de Janeiro, Brazil; 11 Universidade do Estado do Rio de Janeiro, Rio de Janeiro, Brazil; 12 Universidade Estadual Paulista , Universidade Federal do ABC, São Paulo, Brazil; 13 Institute for Nuclear Research and Nuclear Energy, Sofia, Bulgaria; 14 University of Sofia, Sofia, Bulgaria; 15 Beihang University, Beijing, China; 16 Institute of High Energy Physics, Beijing, China; 17 State Key Laboratory of Nuclear Physics and Technology, Peking University, Beijing, China; 18 Universidad de Los Andes, Bogota, Colombia; 19 University of Split, Faculty of Electrical Engineering, Mechanical Engineering and Naval Architecture, Split, Croatia; 20 University of Split, Faculty of Science, Split, Croatia; 21 Institute Rudjer Boskovic, Zagreb, Croatia; 22 University of Cyprus, Nicosia, Cyprus; 23 Charles University, Prague, Czech Republic; 24 Universidad San Francisco de Quito, Quito, Ecuador; 25 Academy of Scientific Research and Technology of the Arab Republic of Egypt, Egyptian Network of High Energy Physics, Cairo, Egypt; 26 National Institute of Chemical Physics and Biophysics, Tallinn, Estonia; 27 Department of Physics, University of Helsinki, Helsinki, Finland; 28 Helsinki Institute of Physics, Helsinki, Finland; 29 Lappeenranta University of Technology, Lappeenranta, Finland; 30 IRFU, CEA, Université Paris-Saclay, Gif-sur-Yvette, France; 31 Laboratoire Leprince-Ringuet, Ecole polytechnique, CNRS/IN2P3, Université Paris-Saclay, Palaiseau, France; 32 Université de Strasbourg, CNRS, IPHC UMR 7178, 67000 Strasbourg, France; 33 Centre de Calcul de l’Institut National de Physique Nucleaire et de Physique des Particules, CNRS/IN2P3 Villeurbanne, France; 34 Université de Lyon, Université Claude Bernard Lyon 1, CNRS-IN2P3, Institut de Physique Nucléaire de Lyon, Villeurbanne, France; 35 Georgian Technical University, Tbilisi, Georgia; 36 Tbilisi State University, Tbilisi, Georgia; 37 RWTH Aachen University, I. Physikalisches Institut, Aachen, Germany; 38 RWTH Aachen University, III. Physikalisches Institut A, Aachen, Germany; 39 RWTH Aachen University, III. Physikalisches Institut B, Aachen, Germany; 40 Deutsches Elektronen-Synchrotron, Hamburg, Germany; 41 University of Hamburg, Hamburg, Germany; 42 Institut für Experimentelle Kernphysik, Karlsruhe, Germany; 43 Institute of Nuclear and Particle Physics (INPP), NCSR Demokritos, Aghia Paraskevi, Greece; 44 National and Kapodistrian University of Athens, Athens, Greece; 45 University of Ioánnina, Ioánnina, Greece; 46 MTA-ELTE Lendület CMS Particle and Nuclear Physics Group, Eötvös Loránd University, Budapest, Hungary; 47 Wigner Research Centre for Physics, Budapest, Hungary; 48 Institute of Nuclear Research ATOMKI, Debrecen, Hungary; 49 Institute of Physics, University of Debrecen, Debrecen, Hungary; 50 Indian Institute of Science (IISc), Bangalore, India; 51 National Institute of Science Education and Research, Bhubaneswar, India; 52 Panjab University, Chandigarh, India; 53 University of Delhi, Delhi, India; 54 Saha Institute of Nuclear Physics, Kolkata, India; 55 Indian Institute of Technology Madras, Madras, India; 56 Bhabha Atomic Research Centre, Mumbai, India; 57 Tata Institute of Fundamental Research-A, Mumbai, India; 58 Tata Institute of Fundamental Research-B, Mumbai, India; 59 Indian Institute of Science Education and Research (IISER), Pune, India; 60 Institute for Research in Fundamental Sciences (IPM), Tehran, Iran; 61 University College Dublin, Dublin, Ireland; 62 INFN Sezione di Bari , Università di Bari , Politecnico di Bari, Bari, Italy; 63 INFN Sezione di Bologna , Università di Bologna, Bologna, Italy; 64 INFN Sezione di Catania , Università di Catania, Catania, Italy; 65 INFN Sezione di Firenze , Università di Firenze, Firenze, Italy; 66 INFN Laboratori Nazionali di Frascati, Frascati, Italy; 67 INFN Sezione di Genova , Università di Genova, Genova, Italy; 68 INFN Sezione di Milano-Bicocca , Università di Milano-Bicocca, Milan, Italy; 69 INFN Sezione di Napoli , Università di Napoli ’Federico II’ , Naples, Italy, Università della Basilicata , Potenza, Italy, Università G. Marconi, Rome, Italy; 70 INFN Sezione di Padova , Università di Padova , Padoa, Italy, Università di Trento, Trento, Italy; 71 INFN Sezione di Pavia , Università di Pavia, Pavia, Italy; 72 INFN Sezione di Perugia , Università di Perugia, Perugia, Italy; 73 INFN Sezione di Pisa , Università di Pisa , Scuola Normale Superiore di Pisa, Pisa, Italy; 74 INFN Sezione di Roma , Università di Roma, Rome, Italy; 75 INFN Sezione di Torino , Università di Torino , Torino, Italy, Università del Piemonte Orientale, Novara, Italy; 76 INFN Sezione di Trieste , Università di Trieste, Trieste, Italy; 77 Kyungpook National University, Taegu, Korea; 78 Chonbuk National University, Chonju, Korea; 79 Chonnam National University, Institute for Universe and Elementary Particles, Kwangju, Korea; 80 Hanyang University, Seoul, Korea; 81 Korea University, Seoul, Korea; 82 Seoul National University, Seoul, Korea; 83 University of Seoul, Seoul, Korea; 84 Sungkyunkwan University, Suwon, Korea; 85 Vilnius University, Vilnius, Lithuania; 86 National Centre for Particle Physics, Universiti Malaya, Kuala Lumpur, Malaysia; 87 Centro de Investigacion y de Estudios Avanzados del IPN, Mexico City, Mexico; 88 Universidad Iberoamericana, Mexico City, Mexico; 89 Benemerita Universidad Autonoma de Puebla, Puebla, Mexico; 90 Universidad Autónoma de San Luis Potosí, San Luis Potosí, Mexico; 91 University of Auckland, Auckland, New Zealand; 92 University of Canterbury, Christchurch, New Zealand; 93 National Centre for Physics, Quaid-I-Azam University, Islamabad, Pakistan; 94 National Centre for Nuclear Research, Swierk, Poland; 95 Institute of Experimental Physics, Faculty of Physics, University of Warsaw, Warsaw, Poland; 96 Laboratório de Instrumentação e Física Experimental de Partículas, Lisbon, Portugal; 97 Joint Institute for Nuclear Research, Dubna, Russia; 98 Petersburg Nuclear Physics Institute, Gatchina (St. Petersburg), Russia; 99 Institute for Nuclear Research, Moscow, Russia; 100 Institute for Theoretical and Experimental Physics, Moscow, Russia; 101 Moscow Institute of Physics and Technology, Moscow, Russia; 102 National Research Nuclear University ’Moscow Engineering Physics Institute’ (MEPhI), Moscow, Russia; 103 P.N. Lebedev Physical Institute, Moscow, Russia; 104 Skobeltsyn Institute of Nuclear Physics, Lomonosov Moscow State University, Moscow, Russia; 105 Novosibirsk State University (NSU), Novosibirsk, Russia; 106 State Research Center of Russian Federation, Institute for High Energy Physics, Protvino, Russia; 107 University of Belgrade, Faculty of Physics and Vinca Institute of Nuclear Sciences, Belgrade, Serbia; 108 Centro de Investigaciones Energéticas Medioambientales y Tecnológicas (CIEMAT), Madrid, Spain; 109 Universidad Autónoma de Madrid, Madrid, Spain; 110 Universidad de Oviedo, Oviedo, Spain; 111 Instituto de Física de Cantabria (IFCA), CSIC-Universidad de Cantabria, Santander, Spain; 112 CERN, European Organization for Nuclear Research, Geneva, Switzerland; 113 Paul Scherrer Institut, Villigen, Switzerland; 114 Institute for Particle Physics, ETH Zurich, Zurich, Switzerland; 115 Universität Zürich, Zurich, Switzerland; 116 National Central University, Chung-Li, Taiwan; 117 National Taiwan University (NTU), Taipei, Taiwan; 118 Chulalongkorn University, Faculty of Science, Department of Physics, Bangkok, Thailand; 119 Cukurova University, Physics Department, Science and Art Faculty, Adana, Turkey; 120 Middle East Technical University, Physics Department, Ankara, Turkey; 121 Bogazici University, Istanbul, Turkey; 122 Istanbul Technical University, Istanbul, Turkey; 123 Institute for Scintillation Materials of National Academy of Science of Ukraine, Kharkov, Ukraine; 124 National Scientific Center, Kharkov Institute of Physics and Technology, Kharkov, Ukraine; 125 University of Bristol, Bristol, UK; 126 Rutherford Appleton Laboratory, Didcot, UK; 127 Imperial College, London, UK; 128 Brunel University, Uxbridge, UK; 129 Baylor University, Waco, USA; 130 Catholic University of America, Washington, USA; 131 The University of Alabama, Tuscaloosa, USA; 132 Boston University, Boston, USA; 133 Brown University, Providence, USA; 134 University of California, Davis, Davis, USA; 135 University of California, Los Angeles, USA; 136 University of California, Riverside, Riverside USA; 137 University of California, San Diego, La Jolla USA; 138 University of California, Santa Barbara - Department of Physics, Santa Barbara, USA; 139 California Institute of Technology, Pasadena, USA; 140 Carnegie Mellon University, Pittsburgh, USA; 141 University of Colorado Boulder, Boulder, USA; 142 Cornell University, Ithaca, USA; 143 Fermi National Accelerator Laboratory, Batavia, USA; 144 University of Florida, Gainesville, USA; 145 Florida International University, Miami, USA; 146 Florida State University, Tallahassee, USA; 147 Florida Institute of Technology, Melbourne, USA; 148 University of Illinois at Chicago (UIC), Chicago, USA; 149 The University of Iowa, Iowa City, USA; 150 Johns Hopkins University, Baltimore, USA; 151 The University of Kansas, Lawrence, USA; 152 Kansas State University, Manhattan, USA; 153 Lawrence Livermore National Laboratory, Livermore, USA; 154 University of Maryland, College Park, USA; 155 Massachusetts Institute of Technology, Cambridge, USA; 156 University of Minnesota, Minneapolis, USA; 157 University of Mississippi, Oxford, USA; 158 University of Nebraska-Lincoln, Lincoln, USA; 159 State University of New York at Buffalo, Buffalo, USA; 160 Northeastern University, Boston, USA; 161 Northwestern University, Evanston, USA; 162 University of Notre Dame, Notre Dame, USA; 163 The Ohio State University, Columbus, USA; 164 Princeton University, Princeton, USA; 165 University of Puerto Rico, Mayaguez, USA; 166 Purdue University, West Lafayette, USA; 167 Purdue University Northwest, Hammond, USA; 168 Rice University, Houston, USA; 169 University of Rochester, Rochester, USA; 170 The Rockefeller University, New York, USA; 171 Rutgers, The State University of New Jersey, Piscataway, USA; 172 University of Tennessee, Knoxville, USA; 173 Texas A&M University, College Station, USA; 174 Texas Tech University, Lubbock, USA; 175 Vanderbilt University, Nashville, USA; 176 University of Virginia, Charlottesville, USA; 177 Wayne State University, Detroit, USA; 178 University of Wisconsin-Madison, Madison, WI USA; 179 CERN, 1211 Geneva 23, Switzerland

## Abstract

A data sample of events from proton–proton collisions with two isolated same-sign leptons, missing transverse momentum, and jets is studied in a search for signatures of new physics phenomena by the CMS Collaboration at the LHC. The data correspond to an integrated luminosity of 35.9$\,\text {fb}^{-\text {1}}$, and a center-of-mass energy of 13$\,\text {TeV}$. The properties of the events are consistent with expectations from standard model processes, and no excess yield is observed. Exclusion limits at 95% confidence level are set on cross sections for the pair production of gluinos, squarks, and same-sign top quarks, as well as top-quark associated production of a heavy scalar or pseudoscalar boson decaying to top quarks, and on the standard model production of events with four top quarks. The observed lower mass limits are as high as 1500$\,\text {GeV}$ for gluinos, 830$\,\text {GeV}$ for bottom squarks. The excluded mass range for heavy (pseudo)scalar bosons is 350–360 (350–410)$\,\text {GeV}$. Additionally, model-independent limits in several topological regions are provided, allowing for further interpretations of the results.

## Introduction

Final states with two leptons of same charge, denoted as same-sign (SS) dileptons, are produced rarely by standard model (SM) processes in proton–proton ($\mathrm{p}\mathrm{p}$) collisions. Because the SM rates of SS dileptons are low, studies of these final states provide excellent opportunities to search for manifestations of physics beyond the standard model (BSM). Over the last decades, a large number of new physics mechanisms have been proposed to extend the SM and address its shortcomings. Many of these can give rise to potentially large contributions to the SS dilepton signature, e.g., the production of supersymmetric (SUSY) particles [1, 2], SS top quarks [3, 4], scalar gluons (sgluons) [5, 6], heavy scalar bosons of extended Higgs sectors [7, 8], Majorana neutrinos [9], and vector-like quarks [10].

In the SUSY framework [11–20], the SS final state can appear in R-parity conserving models through gluino or squark pair production when the decay of each of the pair-produced particles yields one or more $\mathrm{W}$ bosons. For example, a pair of gluinos (which are Majorana particles) can give rise to SS charginos and up to four top quarks, yielding signatures with up to four $\mathrm{W}$ bosons, as well as jets, $\mathrm{b}$ quark jets, and large missing transverse momentum ($E_{\mathrm{T}}^{\text {miss}}$). Similar signatures can also result from the pair production of bottom squarks, subsequently decaying to charginos and top quarks.

While R-parity conserving SUSY models often lead to signatures with large $E_{\mathrm{T}}^{\text {miss}}$, it is also interesting to study final states without significant $E_{\mathrm{T}}^{\text {miss}}$ beyond what is produced by the neutrinos from leptonic $\mathrm{W}$ boson decays. For example, some SM and BSM scenarios can lead to the production of SS or multiple top quark pairs, such as the associated production of a heavy (pseudo)scalar, which subsequently decays to a pair of top quarks. This scenario is realized in Type II two Higgs doublet models (2HDM) where associated production with a single top quark or a $\mathrm{t}\overline{\mathrm{t}}$ pair can in some cases provide a promising window to probe these heavy (pseudo)scalar bosons [21–23].

This paper extends the search for new physics presented in Ref. [24]. We consider final states with two leptons (electrons and muons) of same charge, two or more hadronic jets, and moderate $E_{\mathrm{T}}^{\text {miss}}$. Compared to searches with zero or one lepton, this final state provides enhanced sensitivity to low-momentum leptons and SUSY models with compressed mass spectra. The results are based on an integrated luminosity corresponding to 35.9$\,\text {fb}^{-\text {1}}$ of $\sqrt{s} = 13\,\text {TeV}$ proton–proton collisions collected with the CMS detector at the CERN LHC. Previous LHC searches in the SS dilepton channel have been performed by the ATLAS [25–27] and CMS [24, 28–32] Collaborations. With respect to Ref. [24], the event categorization is extended to take advantage of the increased integrated luminosity, the estimate of rare SM backgrounds is improved, and the (pseudo)scalar boson interpretation is added.

The results of the search are interpreted in a number of specific BSM models discussed in Sect. 2. In addition, model-independent results are also provided in several kinematic regions to allow for further interpretations. These results are given as a function of hadronic activity and of $E_{\mathrm{T}}^{\text {miss}}$, as well as in a set of inclusive regions with different topologies. The full analysis results are also summarized in a smaller set of exclusive regions to be used in combination with the background correlation matrix to facilitate their reinterpretation.

## Background and signal simulation

Monte Carlo (MC) simulations are used to estimate SM background contributions and to estimate the acceptance of the event selection for BSM models. The MadGraph 5_amc@nlo 2.2.2 [33–35] and powheg  v2 [36, 37] next-to-leading order (NLO) generators are used to simulate almost all SM background processes based on the NNPDF3.0 NLO [38] parton distribution functions (PDFs). New physics signal samples, as well as the same-sign $\mathrm{W}^{\pm } \mathrm{W}^{\pm }$ process, are generated with MadGraph 5_amc@nlo at leading order (LO) precision, with up to two additional partons in the matrix element calculations, using the NNPDF3.0 LO [38] PDFs. Parton showering and hadronization, as well as the double-parton scattering production of $\mathrm{W}^{\pm } \mathrm{W}^{\pm }$, are described using the pythia  8.205 generator [39] with the CUETP8M1 tune [40, 41]. The Geant4 package [42] is used to model the CMS detector response for background samples, while the CMS fast simulation package [43] is used for signal samples.

To improve on the MadGraph modeling of the multiplicity of additional jets from initial-state radiation (ISR), MadGraph $\mathrm{t}\overline{\mathrm{t}}$ MC events are reweighted based on the number of ISR jets ($N_J^\mathrm{ISR}$), so as to make the light-flavor jet multiplicity in dilepton $\mathrm{t}\overline{\mathrm{t}}$ events agree with the one observed in data. The same reweighting procedure is applied to SUSY MC events. The reweighting factors vary between 0.92 and 0.51 for $N_J^\mathrm{ISR}$ between 1 and 6. We take one half of the deviation from unity as the systematic uncertainty in these reweighting factors.

**Fig. 1:**
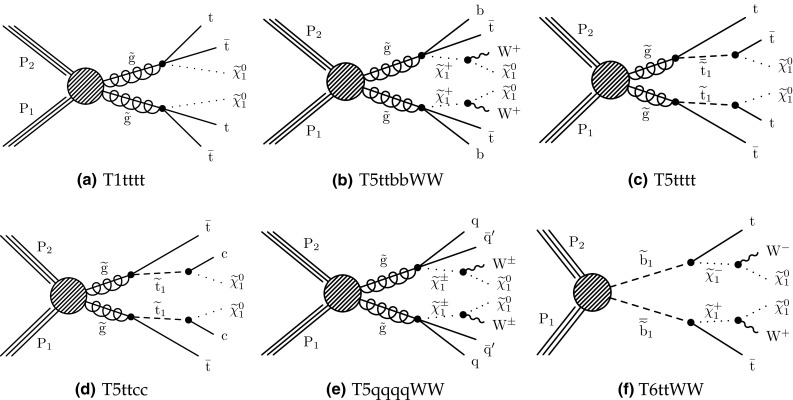
Diagrams illustrating the simplified SUSY models considered in this analysis

**Fig. 2:**
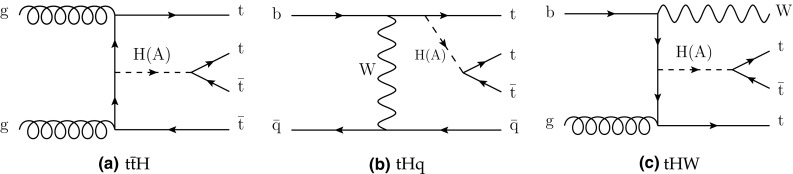
Diagrams for scalar (pseudoscalar) boson production in association with top quarks

The new physics signal models probed by this search are shown in Figs. 1 and 2. In each of the simplified SUSY models [44, 45] of Fig. 1, only two or three new particles have masses sufficiently low to be produced on-shell, and the branching fraction for the decays shown are assumed to be 100%. Gluino pair production models giving rise to signatures with up to four $\mathrm{b}$ quarks and up to four $\mathrm{W}$ bosons are shown in Fig. 1a–e. In these models, the gluino decays to the lightest squark ($\widetilde{\mathrm{g}}\rightarrow \widetilde{\mathrm{q}} \mathrm{q}$), which in turn decays to same-flavor ($\widetilde{\mathrm{q}} \rightarrow \mathrm{q} \widetilde{\chi }^{0}_{1}$) or different-flavor ($\widetilde{\mathrm{q}} \rightarrow \mathrm{q} ' \widetilde{\chi }^\pm _{1}$) quarks. The chargino decays to a $\mathrm{W}$ boson and a neutralino ($\widetilde{\chi }^\pm _{1} \rightarrow \mathrm{W}^{\pm } \widetilde{\chi }^{0}_{1}$), where the $\widetilde{\chi }^{0}_{1}$ escapes detection and is taken to be the lightest SUSY particle (LSP). The first two scenarios considered in Fig. 1a, b include an off-shell third-generation squark ($\widetilde{\mathrm{t}}$ or $\widetilde{\mathrm{b}}$) leading to the three-body decay of the gluino, $\widetilde{\mathrm{g}}\rightarrow \mathrm{t}\overline{\mathrm{t}} \widetilde{\chi }^{0}_{1}$ ($\mathrm{T}1{\mathrm{t} \mathrm{t} \mathrm{t} \mathrm{t}}$) and $\widetilde{\mathrm{g}}\rightarrow \overline{\mathrm{t}}\mathrm{b} \widetilde{\chi }^{+}_{1}$ ($\mathrm{T}5{\mathrm{t} \mathrm{t} \mathrm{b} \mathrm{b}}\mathrm{W}\mathrm{W}$), resulting in events with four $\mathrm{W}$ bosons and four $\mathrm{b}$ quarks. In the $\mathrm{T}5{\mathrm{t} \mathrm{t} \mathrm{b} \mathrm{b}}\mathrm{W}\mathrm{W}$ model, the mass splitting between chargino and neutralino is set to $m_{\widetilde{\chi }^\pm _{1}} - m_{\widetilde{\chi }^{0}_{1}} = 5\,\text {GeV}$, so that two of the $\mathrm{W}$ bosons are produced off-shell and can give rise to low transverse momentum ($p_{\mathrm{T}}$) leptons. The next two models shown (Fig. 1c, d) include an on-shell top squark with different mass splitting between the $\widetilde{\mathrm{t}}$ and the $\widetilde{\chi }^{0}_{1}$, and consequently different decay modes: in the $\mathrm{T}5{\mathrm{t} \mathrm{t} \mathrm{t} \mathrm{t}}$ model the mass splitting is equal to the top quark mass ($m_{\widetilde{\mathrm{t}}} - m_{\widetilde{\chi }^{0}_{1}} = m_{\mathrm{t}}$), favoring the $\widetilde{\mathrm{t}} \rightarrow \mathrm{t} \widetilde{\chi }^{0}_{1}$ decay, while in the $\mathrm{T}5{\mathrm{t} \mathrm{t} \mathrm{c} \mathrm{c}}$ model the mass splitting is only $20\,\text {GeV}$, favoring the flavor changing neutral current $\widetilde{\mathrm{t}} \rightarrow \mathrm{c} \widetilde{\chi }^{0}_{1}$ decay. In Fig. 1e, the decay proceeds through a virtual light-flavor squark, leading to a three-body decay to $\widetilde{\mathrm{g}}\rightarrow \mathrm{q} \mathrm{q} ' \widetilde{\chi }^\pm _{1}$, resulting in a signature with two $\mathrm{W}$ bosons and four light-flavor jets. The two $\mathrm{W}$ bosons can have the same charge, giving rise to SS dileptons. This model, $\mathrm{T}5{\mathrm{q} \mathrm{q} \mathrm{q} \mathrm{q}}\mathrm{W}\mathrm{W}$, is studied as a function of the gluino and $\widetilde{\chi }^{0}_{1}$ mass, with two different assumptions for the chargino mass: $m_{\widetilde{\chi }^\pm _{1}} = 0.5(m_{\widetilde{\mathrm{g}}} + m_{\widetilde{\chi }^{0}_{1}})$, producing mostly on-shell $\mathrm{W}$ bosons, and $m_{\widetilde{\chi }^\pm _{1}} = m_{\widetilde{\chi }^{0}_{1}}+20\,\text {GeV}$, producing off-shell $\mathrm{W}$ bosons. Finally, Fig. 1f shows a model of bottom squark production followed by the $\widetilde{\mathrm{b}} \rightarrow \mathrm{t} \widetilde{\chi }^\pm _{1}$ decay, resulting in two $\mathrm{b}$ quarks and four $\mathrm{W}$ bosons. This model, $\mathrm{T}6{\mathrm{t} \mathrm{t}}\mathrm{W}\mathrm{W}$, is studied as a function of the $\widetilde{\mathrm{b}}$ and $\widetilde{\chi }^\pm _{1}$ masses, keeping the $\widetilde{\chi }^{0}_{1}$ mass at 50$\,\text {GeV}$, resulting in two of the $\mathrm{W}$ bosons being produced off-shell when the $\widetilde{\chi }^\pm _{1}$ and $\widetilde{\chi }^{0}_{1}$ masses are close. The production cross sections for SUSY models are calculated at NLO plus next-to-leading logarithmic (NLL) accuracy [46–51].

The processes shown in Fig. 2, $\mathrm{t}\overline{\mathrm{t}} \mathrm{H}$, $\mathrm{t} \mathrm{H} \mathrm{q}$, and $\mathrm{t} \mathrm{W}\mathrm{H}$, represent the top quark associated production of a scalar ($\mathrm{H}$) or a pseudoscalar ($\mathrm{A}$). The subsequent decay of the (pseudo)scalar to a pair of top quarks then gives rise to final states including a total of three or four top quarks. For the purpose of interpretation, we use LO cross sections for the production of a heavy Higgs boson in the context of the Type II 2HDM of Ref. [23]. The mass of the new particle is varied in the range [350, 550]$\,\text {GeV}$, where the lower mass boundary is chosen in such a way as to allow the decay of the (pseudo)scalar into on-shell top quarks.

## The CMS detector and event reconstruction

The central feature of the CMS detector is a superconducting solenoid of 6$\,\text {m}$ internal diameter, providing a magnetic field of 3.8$\,\text {T}$. Within the solenoid volume are a silicon pixel and strip tracker, a lead tungstate crystal electromagnetic calorimeter (ECAL), and a brass and scintillator hadron calorimeter (HCAL), each composed of a barrel and two endcap sections. Forward calorimeters extend the pseudorapidity ($\eta$) coverage provided by the barrel and endcap detectors. Muons are measured in gas-ionization detectors embedded in the steel flux-return yoke outside the solenoid. A more detailed description of the CMS detector, together with a definition of the coordinate system used and the relevant kinematic variables, can be found in Ref. [52].

Events of interest are selected using a two-tiered trigger system [53]. The first level (L1), composed of custom hardware processors, uses information from the calorimeters and muon detectors to select events at a rate of around 100$\,\text {kHz}$ within a time interval of less than 4 $\,\upmu \text {s}$. The second level, known as the high-level trigger (HLT), consists of a farm of processors running a version of the full event reconstruction software optimized for fast processing, and reduces the event rate to less than 1$\,\text {kHz}$ before data storage.

Events are processed using the particle-flow (PF) algorithm [54, 55], which reconstructs and identifies each individual particle with an optimized combination of information from the various elements of the CMS detector. The energy of photons is directly obtained from the ECAL measurement. The energy of electrons is determined from a combination of the electron momentum at the primary interaction vertex as determined by the tracker, the energy of the corresponding ECAL cluster, and the energy sum of all bremsstrahlung photons spatially compatible with the electron track [56]. The energy of muons is obtained from the curvature of the corresponding track, combining information from the silicon tracker and the muon system [57]. The energy of charged hadrons is determined from a combination of their momentum measured in the tracker and the matching ECAL and HCAL energy deposits, corrected for the response function of the calorimeters to hadronic showers. Finally, the energy of neutral hadrons is obtained from the corresponding corrected ECAL and HCAL energy.

Hadronic jets are clustered from neutral PF candidates and charged PF candidates associated with the primary vertex, using the anti-$k_{\mathrm{T}}$ algorithm [58, 59] with a distance parameter $R = \sqrt{\smash [b]{ (\Delta \eta )^2 + (\Delta \phi )^2} }$ of 0.4. Jet momentum is determined as the vectorial sum of all PF candidate momenta in the jet. An offset correction is applied to jet energies to take into account the contribution from additional proton–proton interactions (pileup) within the same or nearby bunch crossings. Jet energy corrections are derived from simulation, and are improved with in situ measurements of the energy balance in dijet and photon+jet events [60, 61]. Additional selection criteria are applied to each event to remove spurious jet-like features originating from isolated noise patterns in certain HCAL regions. Jets originating from $\mathrm{b}$ quarks are identified (b tagged) using the medium working point of the combined secondary vertex algorithm CSVv2 [62]. The missing transverse momentum vector ${\vec p}_{\mathrm{T}}^{\text {miss}}$ is defined as the projection on the plane perpendicular to the beams of the negative vector sum of the momenta of all reconstructed PF candidates in an event [63]. Its magnitude is referred to as $E_{\mathrm{T}}^{\text {miss}}$. The sum of the transverse momenta of all jets in an event is referred to as $H_{\mathrm{T}}$.

## Event selection and search strategy

The event selection and the definition of the signal regions (SRs) follow closely the analysis strategy established in Ref. [24]. With respect to the previous search, the general strategy has remained unchanged. We target, in a generic way, new physics signatures that result in SS dileptons, hadronic activity, and $E_{\mathrm{T}}^{\text {miss}}$, by subdividing the event sample into several SRs sensitive to a variety of new physics models. The number of SRs was increased to take advantage of the larger integrated luminosity. Table 1 summarizes the basic kinematic requirements for jets and leptons (further details, including the lepton identification and isolation requirements, can be found in Ref. [24]).

**Table 1:** Kinematic requirements for leptons and jets. Note that the $p_{\mathrm{T}}$ thresholds to count jets and b-tagged jets are different

Object	$p_{\mathrm{T}}$ ($\,\text {GeV}$)	$|\eta |$
Electrons	>15	$$2.5 $$
Muons	>10	$$2.4 $$
Jets	>40	$$2.4 $$
b-tagged jets	>25	$$2.4 $$

Events are selected using triggers based on two sets of HLT algorithms, one simply requiring two leptons, and one additionally requiring $H_{\mathrm{T}} > 300\,\text {GeV}$. The $H_{\mathrm{T}}$ requirement allows for the lepton isolation requirement to be removed and for the lepton $p_{\mathrm{T}}$ thresholds to be set to 8$\,\text {GeV}$ for both leptons, while in the pure dilepton trigger the leading and subleading leptons are required to have $p_{\mathrm{T}} > 23~(17)\,\text {GeV}$ and $p_{\mathrm{T}} > 12~(8)\,\text {GeV}$, respectively, for electrons (muons). Based on these trigger requirements, leptons are classified as high ($p_{\mathrm{T}} > 25\,\text {GeV}$) and low ($10< p_{\mathrm{T}} < 25\,\text {GeV}$) momentum, and three analysis regions are defined: high-high (HH), high-low (HL), and low-low (LL).

The baseline selection used in this analysis requires at least one SS lepton pair with an invariant mass above 8$\,\text {GeV}$, at least two jets, and $E_{\mathrm{T}}^{\text {miss}} > 50\,\text {GeV}$. To reduce Drell–Yan backgrounds, events are rejected if an additional loose lepton forms an opposite-sign same-flavor pair with one of the two SS leptons, with an invariant mass less than 12$\,\text {GeV}$ or between 76 and 106$\,\text {GeV}$. Events passing the baseline selection are then divided into SRs to separate the different background processes and to maximize the sensitivity to signatures with different jet multiplicity ($N_\text {jets}$), flavor ($N_{\mathrm{b}}$), visible and invisible energy ($H_{\mathrm{T}}$ and $E_{\mathrm{T}}^{\text {miss}}$), and lepton momentum spectra (the HH/HL/LL categories mentioned previously). The $m_\mathrm{T}^{\text {min}}$ variable is defined as the smallest of the transverse masses constructed between ${\vec p}_{\mathrm{T}}^{\text {miss}}$ and each of the leptons. This variable features a cutoff near the $\mathrm{W}$ boson mass for processes with only one prompt lepton, so it is used to create SRs where the nonprompt lepton background is negligible. To further improve sensitivity, several regions are split according to the charge of the leptons ($++$ or −−), taking advantage of the charge asymmetry of SM backgrounds, such as $\mathrm{t}\overline{\mathrm{t}} \mathrm{W}$ or $\mathrm{W}$ $\mathrm{Z}$, with a single $\mathrm{W}$ boson produced in $\mathrm{p}\mathrm{p}$ collisions. Only signal regions dominated by such backgrounds and with a sufficient predicted yield are split by charge. In the HH and HL categories, events in the tail regions $H_{\mathrm{T}} > 1125\,\text {GeV}$ or $E_{\mathrm{T}}^{\text {miss}} > 300\,\text {GeV}$ are inclusive in $N_\text {jets}$, $N_{\mathrm{b}}$, and $m_\mathrm{T}^{\text {min}}$ in order to ensure a reasonable yield of events in these SRs. The exclusive SRs resulting from this classification are defined in Tables 2, 3 and 4.

The lepton reconstruction and identification efficiency is in the range of 45–70% (70–90%) for electrons (muons) with $p_{\mathrm{T}} >25\,\text {GeV}$, increasing as a function of $p_{\mathrm{T}}$ and converging to the maximum value for $p_{\mathrm{T}} >60\,\text {GeV}$. In the low-momentum regime, $15<p_{\mathrm{T}} <25\,\text {GeV}$ for electrons and $10<p_{\mathrm{T}} <25\,\text {GeV}$ for muons, the efficiencies are 40% for electrons and 55% for muons. The lepton trigger efficiency for electrons is in the range of 90–98%, converging to the maximum value for $p_{\mathrm{T}} >30\,\text {GeV}$, and around 92% for muons. The chosen b tagging working point results in approximately a 70% efficiency for tagging a $\mathrm{b}$ quark jet and a <1% mistagging rate for light-flavor jets in $\mathrm{t}\overline{\mathrm{t}}$ events [62]. The efficiencies of the $H_{\mathrm{T}}$ and $E_{\mathrm{T}}^{\text {miss}}$ requirements are mostly determined by the jet energy and $E_{\mathrm{T}}^{\text {miss}}$ resolutions, which are discussed in Refs. [60, 61, 64].

**Table 2:** Signal region definitions for the HH selection. Regions split by charge are indicated with (++) and ($-$ $-$). All unlabeled region are included in the SR above them, for example the unlabeled regions between SR3 and SR11 are included in SR3, with the exception of the regions to the right of SR42-45, which are included in those regions

$N_{\mathrm{b}}$	$m_\mathrm{T}^{\text {min}}$ ($\text {GeV}$ )	$E_{\mathrm{T}}^{\text {miss}}$ ($\text {GeV}$ )	$N_\text {jets}$	$H_{\mathrm{T}} < 300\,\text {GeV}$	$H_{\mathrm{T}} \in [300, 1125]\,\,\text {GeV}$	$H_{\mathrm{T}} \in [1125, 1300]\,\,\text {GeV}$	$H_{\mathrm{T}} \in [1300, 1600]\,\,\text {GeV}$	$H_{\mathrm{T}} > 1600\,\text {GeV}$
0	<120	$50{-}200$	$2{-}4$	SR1	SR2	SR46 (++)/SR47 ($-$ $-$)	SR48 (++)/SR49 ($-$ $-$)	SR50 (++)/SR51 ($-$ $-$)
			$\ge$5	SR3	SR4			
		$200{-}300$	2–4		SR5 (++)/SR6 ($-$ $-$)			
			$\ge$5		SR7			
	>120	$50{-}200$	2–4		SR8 (++)/SR9 ($-$ $-$)			
			$\ge$5		SR10			
		$200{-}300$	2–4					
			$\ge$5					
1	<120	$50{-}200$	2–4	SR11	SR12			
			$\ge$5	SR13 (++)/SR14 ($-$ $-$)	SR15 (++)/SR16 ($-$ $-$)			
		$200{-}300$	2–4		SR17 (++)/SR18 ($-$ $-$)			
			$\ge$5		SR19			
	>120	$50{-}200$	2–4		SR20 (++)/SR21 ($-$ $-$)			
			$\ge$5		SR22			
		$200{-}300$	2–4					
			$\ge$5					
2	<120	$50{-}200$	2–4	SR23	SR24			
			$\ge$5	SR25 (++)/SR26 ($-$ $-$)	SR27 (++)/SR28 ($-$ $-$)			
		$200{-}300$	2–4		SR29 (++)/SR30 ($-$ $-$)			
			$\ge$5		SR31			
	>120	$50{-}200$	2–4		SR32 (++)/SR33 ($-$ $-$)			
			$\ge$5		SR34			
		$200{-}300$	2–4					
			$\ge$5					
$\ge$3	<120	$50{-}200$	$\ge$2	SR35 (++)/SR36 ($-$ $-$)	SR37 (++)/SR38 ($-$ $-$)			
		$200{-}300$			SR39			
	>120	$50{-}300$	$\ge$2	SR40	SR41			
Inclusive	Inclusive	$300{-}500$	$\ge$2	–	SR42 (++)/SR43 ($-$ $-$)			
		>500		–	SR44 (++)/SR45 ($-$ $-$)			

**Table 3:** Signal region definitions for the HL selection. Regions split by charge are indicated with ($++$) and ($-$ $-$). All unlabeled region are included in the SR above them, for example the unlabeled regions between SR3 and SR8 are included in SR3, with the exception of the regions to the right of SR34-37, which are included in those regions

$N_{\mathrm{b}}$	$m_\mathrm{T}^{\text {min}}$ ($\text {GeV}$ )	$E_{\mathrm{T}}^{\text {miss}}$ ($\text {GeV}$ )	$N_\text {jets}$	$H_{\mathrm{T}} < 300\,\text {GeV}$	$H_{\mathrm{T}} \in [300, 1125]\,\text {GeV}$	$H_{\mathrm{T}} \in [1125, 1300]\,\text {GeV}$	$H_{\mathrm{T}} > 1300\,\text {GeV}$
0	<120	$50{-}200$	2-4	SR1	SR2	SR38 (++)/SR39 ($-$ $-$)	SR40 (++) / SR41 ($-$ $-$)
			$\ge$5	SR3	SR4		
		$200{-}300$	2–4		SR5 (++)/SR6 ($-$ $-$)		
			$\ge$5		SR7		
1	<120	$50{-}200$	2-4	SR8	SR9		
			$\ge$5	SR10 (++)/SR11 ($-$ $-$)	SR12 (++)/SR13 ($-$ $-$)		
		$200{-}300$	2–4		SR14 (++)/SR15 ($-$ $-$)		
			$\ge$5		SR16 (++)/SR17 ($-$ $-$)		
2	<120	$50{-}200$	2–4	SR18	SR19		
			$\ge$5	SR20 (++)/SR21 ($-$ $-$)	SR22 (++)/SR23 ($-$ $-$)		
		$200{-}300$	2–4		SR24 (++)/SR25 ($-$ $-$)		
			$\ge$5		SR26		
$\ge$3	<120	$50{-}200$	$\ge$2	SR27 (++)/SR28 ($-$ $-$)	SR29 (++)/SR30 ($-$ $-$)		
		$200{-}300$			SR31		
Inclusive	>120	$50{-}300$	$\ge$2	SR32	SR33		
Inclusive	Inclusive	$300{-}500$	$\ge$2	–	SR34 (++)/SR35 ($-$ $-$)		
		>500		–	SR36 (++)/SR37 ($-$ $-$)		

**Table 4:** Signal region definitions for the LL selection. All SRs in this category require $N_\text {jets} \ge 2$

$N_{\mathrm{b}}$	$m_\mathrm{T}^{\text {min}}$ ($\text {GeV}$ )	$H_{\mathrm{T}}$ ($\text {GeV}$ )	$E_{\mathrm{T}}^{\text {miss}} \in [50, 200]\,\text {GeV}$	$E_{\mathrm{T}}^{\text {miss}} > 200\,\text {GeV}$
0	<120	>300	SR1	SR2
1			SR3	SR4
2			SR5	SR6
$\ge$3			SR7	
Inclusive	>120		SR8	

## Backgrounds

Standard model background contributions arise from three sources: processes with prompt SS dileptons, mostly relevant in regions with high $E_{\mathrm{T}}^{\text {miss}}$ or $H_{\mathrm{T}}$; events with a nonprompt lepton, dominating the overall final state; and opposite-sign dilepton events with a charge-misidentified lepton, the smallest contribution. In this paper we use the shorthand “nonprompt leptons” to refer to electrons or muons from the decays of heavy- or light-flavor hadrons, hadrons misidentified as leptons, or electrons from conversions of photons in jets.

Several categories of SM processes that result in the production of electroweak bosons can give rise to an SS dilepton final state. These include production of multiple bosons in the same event (prompt photons, $\mathrm{W}$, $\mathrm{Z}$, and Higgs bosons), as well as single-boson production in association with top quarks. Among these SM processes, the dominant ones are $\mathrm{W}$ $\mathrm{Z}$, $\mathrm{t}\overline{\mathrm{t}} \mathrm{W}$, and $\mathrm{t}\overline{\mathrm{t}} \mathrm{Z}$ production, followed by the $\mathrm{W}^{\pm }\mathrm{W}^{\pm }$ process. The remaining SM processes are grouped into two categories, “Rare” (including $\mathrm{Z} \mathrm{Z}$, $\mathrm{W}$ $\mathrm{W}$ $\mathrm{Z}$, $\mathrm{W}\mathrm{Z} \mathrm{Z}$, $\mathrm{Z} \mathrm{Z} \mathrm{Z}$, $\mathrm{t} \mathrm{W}\mathrm{Z}$, $\mathrm{t} \mathrm{Z} \mathrm{q}$, as well as $\mathrm{t}\overline{\mathrm{t}} \mathrm{t}\overline{\mathrm{t}}$ and double parton scattering) and “X+${\upgamma }$” (including $\mathrm{W}{\upgamma }$, $\mathrm{Z} {\upgamma }$, $\mathrm{t}\overline{\mathrm{t}} {\upgamma }$, and $\mathrm{t} {\upgamma }$). The expected yields from these SM backgrounds are estimated from simulation, accounting for both the theoretical and experimental uncertainties discussed in Sect. 6.

For the $\mathrm{W}$ $\mathrm{Z}$ and $\mathrm{t}\overline{\mathrm{t}} \mathrm{Z}$ backgrounds, a three-lepton (3L) control region in data is used to scale the simulation, based on a template fit to the distribution of the number of b jets. The 3L control region requires at least two jets, $E_{\mathrm{T}}^{\text {miss}} > 30\,\text {GeV}$, and three leptons, two of which must form an opposite-sign same-flavor pair with an invariant mass within $15\,\text {GeV}$ of the $\mathrm{Z}$ boson mass. In the fit to data, the normalization and shapes of all the components are allowed to vary according to experimental and theoretical uncertainties. The scale factors obtained from the fit in the phase space of the 3L control region are $1.26 \pm 0.09$ for the $\mathrm{W}$ $\mathrm{Z}$ process, and $1.14 \pm 0.30$ for the $\mathrm{t}\overline{\mathrm{t}} \mathrm{Z}$ process.

The nonprompt lepton background, which is largest for regions with low $m_\mathrm{T}^{\text {min}}$ and low $H_{\mathrm{T}}$, is estimated by the “tight-to-loose” method, which was employed in several previous versions of the analysis [28–32], and significantly improved in the latest version [24] to account for the kinematics and flavor of the parent parton of the nonprompt lepton. The tight-to-loose method uses two control regions, the measurement region and the application region. The measurement region consists of a sample of single-lepton events enriched in nonprompt leptons by requirements on $E_{\mathrm{T}}^{\text {miss}}$ and transverse mass that suppress the $\mathrm{W}\rightarrow \ell \nu$ contribution. This sample is used to extract the probability for a nonprompt lepton that satisfies the loose selection to also satisfy the tight selection. This probability ($\epsilon _\mathrm{TL}$) is calculated as a function of lepton $p_{\mathrm{T}} ^\text {corr}$ (defined below) and $\eta$, separately for electrons and muons, and separately for lepton triggers with and without an isolation requirement. The application region is a SS dilepton region where both of the leptons satisfy the loose selection but at least one of them fails the tight selection. This region is subsequently divided into a set of subregions with the exact same kinematic requirements as those in the SRs. Events in the subregions are weighted by a factor $\epsilon _\mathrm{TL} / (1-\epsilon _\mathrm{TL})$ for each lepton in the event failing the tight requirement. The nonprompt background in each SR is then estimated as the sum of the event weights in the corresponding subregion. The $p_{\mathrm{T}} ^\text {corr}$ parametrization, where $p_{\mathrm{T}} ^\text {corr}$ is defined as the lepton $p_{\mathrm{T}}$ plus the energy in the isolation cone exceeding the isolation threshold value, is chosen because of its correlation with the parent parton $p_{\mathrm{T}}$, improving the stability of the $\epsilon _\mathrm{TL}$ values with respect to the sample kinematics. To improve the stability of the $\epsilon _\mathrm{TL}$ values with respect to the flavor of the parent parton, the loose electron selection is adopted. This selection increases the number of nonprompt electrons from the fragmentation and decay of light-flavor partons, resulting in $\epsilon _\mathrm{TL}$ values similar to those from heavy-flavor parent partons.

The prediction from the tight-to-loose method is cross-checked using an alternative method based on the same principle, similar to that described in Ref. [65]. In this cross-check, which aims to remove kinematic differences between measurement and application regions, the measurement region is obtained from SS dilepton events where one of the leptons fails the impact parameter requirement. With respect to the nominal method, the loose lepton definition is adapted to reduce the effect of the correlation between isolation and impact parameter. The predictions of the two methods are found to be consistent within systematic uncertainties.

Charge misidentification of electrons is a small background that can arise from severe bremsstrahlung in the tracker material. Simulation-based studies with tight leptons indicate that the muon charge misidentification probability is negligible, while for electrons it ranges between $10^{-5}$ and $10^{-3}$. The charge misidentification background is estimated from data using an opposite-sign control region for each SS SR, scaling the control region yield by the charge misidentification probability measured in simulation. A low-$E_{\mathrm{T}}^{\text {miss}}$ control region, with $\mathrm{e}^+\mathrm{e}^-$ pairs in the $\mathrm{Z}$ boson mass window, is used to cross-check the MC prediction for the misidentification probability, both inclusively and – where the number of events in data allows it – as a function of electron $p_{\mathrm{T}}$ and $\eta$.

## Systematic uncertainties

Several sources of systematic uncertainty affect the predicted yields for signal and background processes, as summarized in Table 5. Experimental uncertainties are based on measurements in data of the trigger efficiency, the lepton identification efficiency, the b tagging efficiency [62], the jet energy scale, and the integrated luminosity [66], as well as on the inelastic cross section value affecting the pileup rate. Theoretical uncertainties related to unknown higher-order effects are estimated by varying simultaneously the factorization and renormalization scales by a factor of two, while uncertainties in the PDFs are obtained using replicas of the NNPDF3.0 set [38].

Experimental and theoretical uncertainties affect both the overall yield (normalization) and the relative population (shape) across SRs, and they are taken into account for all signal samples as well as for the samples used to estimate the main prompt SS dilepton backgrounds: $\mathrm{W}$ $\mathrm{Z}$, $\mathrm{t}\overline{\mathrm{t}} \mathrm{W}$, $\mathrm{t}\overline{\mathrm{t}} \mathrm{Z}$, $\mathrm{W}^{\pm } \mathrm{W}^{\pm }$. For the $\mathrm{W}$ $\mathrm{Z}$ and $\mathrm{t}\overline{\mathrm{t}} \mathrm{Z}$ backgrounds, the control region fit results are used for the normalization, so these uncertainties are only taken into account for the shape of the backgrounds. For the smallest background samples, Rare and X+${\upgamma }$, a 50% uncertainty is assigned in place of the scale and PDF variations.

The normalization and the shapes of the nonprompt lepton and charge misidentification backgrounds are estimated from control regions in data. In addition to the statistical uncertainties from the control region yields, dedicated systematic uncertainties are associated with the methods used in this estimate. For the nonprompt lepton background, a 30% uncertainty (increased to 60% for electrons with $p_{\mathrm{T}} > 50\,\text {GeV}$) accounts for the performance of the method in simulation and for the differences in the two alternative methods described in Sect. 5. In addition, the uncertainty in the prompt lepton yield in the measurement region, relevant when estimating $\epsilon _\mathrm{TL}$ for high-$p_{\mathrm{T}}$ leptons, results in a 1–30% effect on the estimate. For the charge misidentification background, a 20% uncertainty is assigned to account for possible mismodeling of the charge misidentification rate in simulation.

**Table 5:** Summary of the sources of uncertainty and their effect on the yields of different processes in the SRs. The first eight uncertainties are related to experimental and theoretical factors for processes estimated using simulation, while the last four uncertainties are assigned to processes whose yield is estimated from data. The first seven uncertainties also apply to signal samples. Reported values are representative for the most relevant signal regions

Source	Typical uncertainty (%)
Integrated luminosity	2.5
Lepton selection	$4{-}10$
Trigger efficiency	$2{-}7$
Pileup	$0-6$
Jet energy scale	$1{-}15$
$\mathrm{b}$ tagging	$1{-}15$
Simulated sample size	$1{-}10$
Scale and PDF variations	$10{-}20$
$\mathrm{W}$ $\mathrm{Z}$ (normalization)	12
$\mathrm{t}\overline{\mathrm{t}} \mathrm{Z}$ (normalization)	30
Nonprompt leptons	$30{-}60$
Charge misidentification	20

## Results and interpretation

**Fig. 3:**
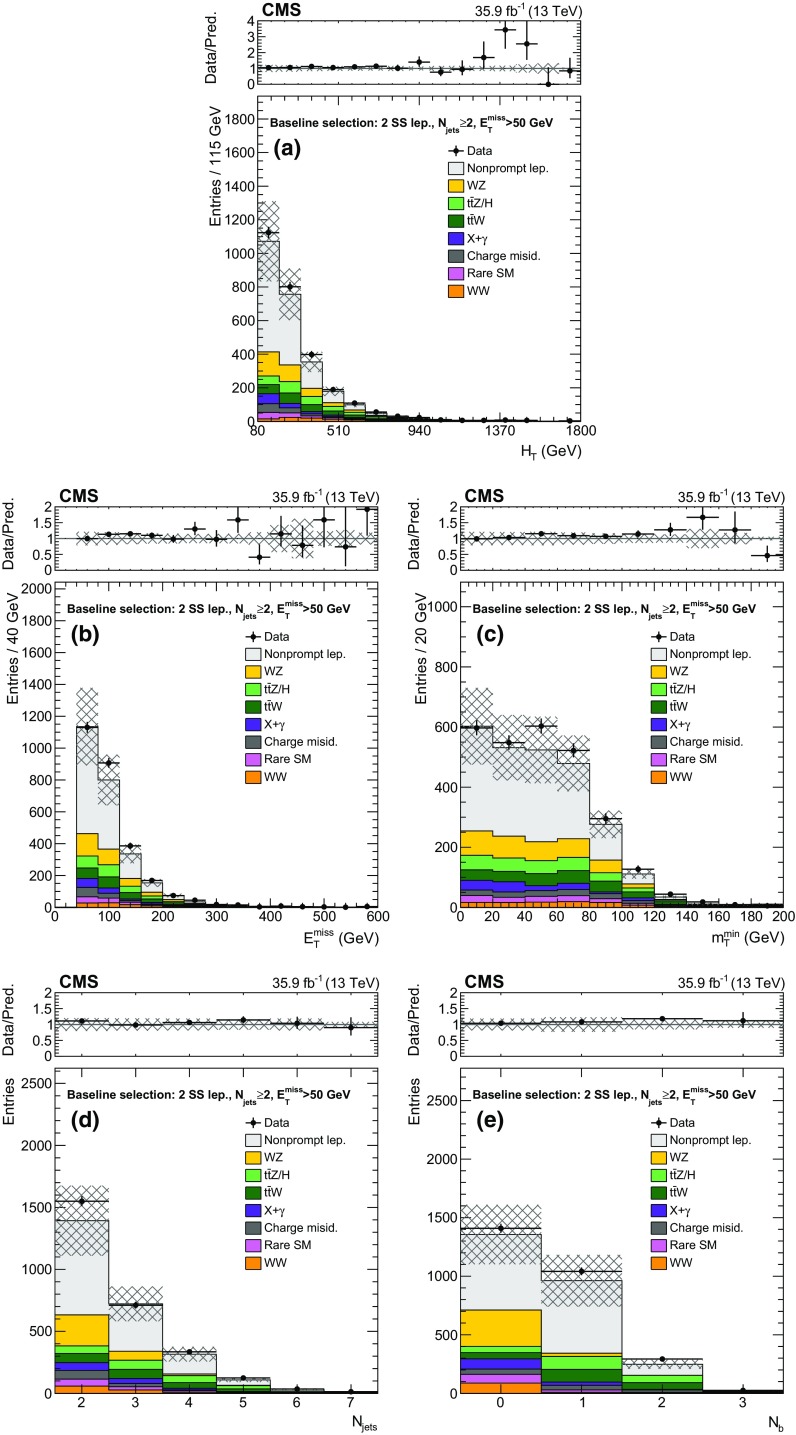
Distributions of the main analysis variables: $H_{\mathrm{T}}$  (**a**), $E_{\mathrm{T}}^{\text {miss}}$  (**b**), $m_\mathrm{T}^{\text {min}}$  (**c**), $N_\text {jets}$  (**d**), and $N_{\mathrm{b}}$  (**e**), after the baseline selection requiring a pair of SS leptons, two jets, and $E_{\mathrm{T}}^{\text {miss}} > 50\,\text {GeV}$. The last bin includes the overflow events and the *hatched area* represents the total uncertainty in the background prediction. The *upper panels* show the ratio of the observed event yield to the background prediction

**Fig. 4:**
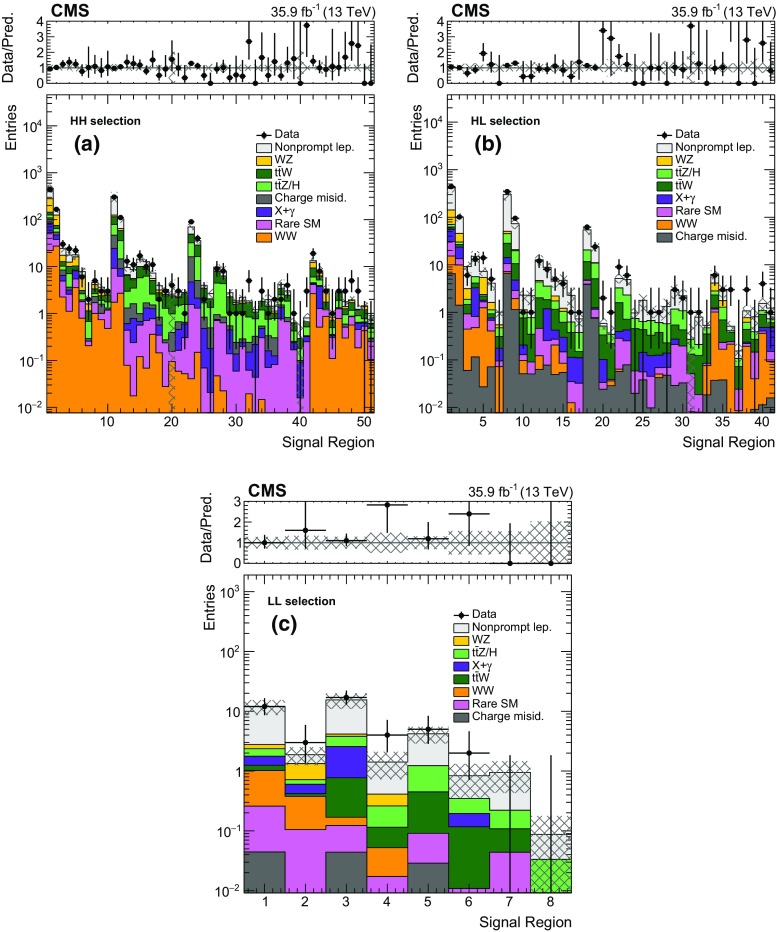
Event yields in the HH (**a**), HL (**b**), and LL (**c**) signal regions. The *hatched area* represents the total uncertainty in the background prediction. The *upper panels* show the ratio of the observed event yield to the background prediction

A comparison between observed yields and the SM background prediction is shown in Fig. 3 for the kinematic variables used to define the analysis SRs: $H_{\mathrm{T}}$, $E_{\mathrm{T}}^{\text {miss}}$, $m_\mathrm{T}^{\text {min}}$, $N_\text {jets}$, and $N_{\mathrm{b}}$. The distributions are shown after the baseline selection defined in Sect. 4. The full results of the search in each SR are shown in Fig. 4 and Table 6. The SM predictions are generally consistent with the data. The largest deviations are seen in HL SR 36 and 38, with a local significance, taking these regions individually or combining them with other regions adjacent in phase space, that does not exceed 2 standard deviations.

These results are used to probe the signal models discussed in Sect. 2: simplified SUSY models, (pseudo)scalar boson production, four top quark production, and SS top quark production. We also interpret the results as model-independent limits as a function of $H_{\mathrm{T}}$ and $E_{\mathrm{T}}^{\text {miss}}$. With the exception of the new (pseudo)scalar boson limits, the results can be compared to the previous version of the analysis [24], showing significant improvements due to the increase in the integrated luminosity and the optimization of SR definitions.

To obtain exclusion limits at the 95% confidence level (CL), the results from all SRs – including signal and background uncertainties and their correlations – are combined using an asymptotic formulation of the modified frequentist CL$_\mathrm{s}$ criterion [67–70]. When testing a model, all new particles not included in the specific model are considered too heavy to take part in the interaction. To convert cross section limits into mass limits, the signal cross sections specified in Sect. 2 are used.

**Table 6:** Number of expected background and observed events in different SRs in this analysis

	HH regions		HL regions		LL regions	
	Expected SM	Observed	Expected SM	Observed	Expected SM	Observed
SR1	468 ± 98	435	419 ± 100	442	12.0 ± 3.9	12
SR2	162 ± 25	166	100 ± 20	101	1.88 ± 0.62	3
SR3	24.4 ± 5.4	30	9.2 ± 2.4	6	15.5 ± 4.7	17
SR4	17.6 ± 3.0	24	15.0 ± 4.5	13	1.42 ± 0.69	4
SR5	17.8 ± 3.9	22	7.3 ± 1.5	14	4.2 ± 1.4	5
SR6	7.8 ± 1.5	6	4.1 ± 1.2	5	0.84 ± 0.48	2
SR7	1.96 ± 0.47	2	1.01 ± 0.28	0	0.95 ± 0.52	0
SR8	4.58 ± 0.81	5	300 ± 82	346	0.09 ± 0.07	0
SR9	3.63 ± 0.75	3	73 ± 17	95		
SR10	2.82 ± 0.56	3	2.30 ± 0.61	1		
SR11	313 ± 87	304	2.24 ± 0.87	1		
SR12	104 ± 20	111	12.8 ± 3.3	12		
SR13	9.5 ± 1.9	13	8.9 ± 2.3	8		
SR14	8.7 ± 2.0	11	4.5 ± 1.3	5		
SR15	14.4 ± 2.9	17	4.7 ± 1.6	4		
SR16	12.7 ± 2.6	10	2.3 ± 1.1	1		
SR17	7.3 ± 1.2	11	0.73 ± 0.29	1		
SR18	3.92 ± 0.79	2	54 ± 12	62		
SR19	3.26 ± 0.74	3	23.7 ± 4.9	24		
SR20	2.6 ± 2.7	4	0.59 ± 0.17	2		
SR21	3.02 ± 0.75	3	0.34 ± 0.20	1		
SR22	2.80 ± 0.57	1	5.2 ± 1.2	9		
SR23	70 ± 12	90	4.9 ± 1.4	6		
SR24	35.7 ± 5.9	40	0.97 ± 0.27	0		
SR25	3.99 ± 0.73	2	1.79 ± 0.74	0		
SR26	2.68 ± 0.80	0	1.01 ± 0.27	1		
SR27	9.7 ± 1.8	9	1.03 ± 0.44	1		
SR28	7.9 ± 2.5	8	1.33 ± 0.61	0		
SR29	2.78 ± 0.58	1	2.89 ± 0.99	3		
SR30	1.86 ± 0.38	1	2.24 ± 0.79	2		
SR31	2.20 ± 0.54	1	0.27 ± 0.30	1		
SR32	1.85 ± 0.39	5	0.79 ± 0.33	1		
SR33	1.20 ± 0.32	0	0.53 ± 0.13	0		
SR34	1.81 ± 0.42	3	6.3 ± 1.3	6		
SR35	1.98 ± 0.61	1	2.92 ± 0.87	3		
SR36	1.43 ± 0.37	2	0.51 ± 0.15	3		
SR37	4.2 ± 1.3	2	0.15 ± 0.07	0		
SR38	3.04 ± 0.68	4	1.07 ± 0.33	3		
SR39	0.63 ± 0.17	1	0.81 ± 0.47	0		
SR40	0.29 ± 0.34	0	1.54 ± 0.50	4		
SR41	0.80 ± 0.22	3	1.23 ± 0.53	1		
SR42	13.4 ± 1.9	19				
SR43	8.0 ± 3.0	8				
SR44	3.33 ± 0.74	3				
SR45	0.94 ± 0.26	1				
SR46	2.92 ± 0.50	3				
SR47	1.78 ± 0.42	3				
SR48	1.95 ± 0.39	5				
SR49	1.23 ± 0.30	3				
SR50	1.46 ± 0.31	0				
SR51	0.74 ± 0.18	0				

**Fig. 5:**
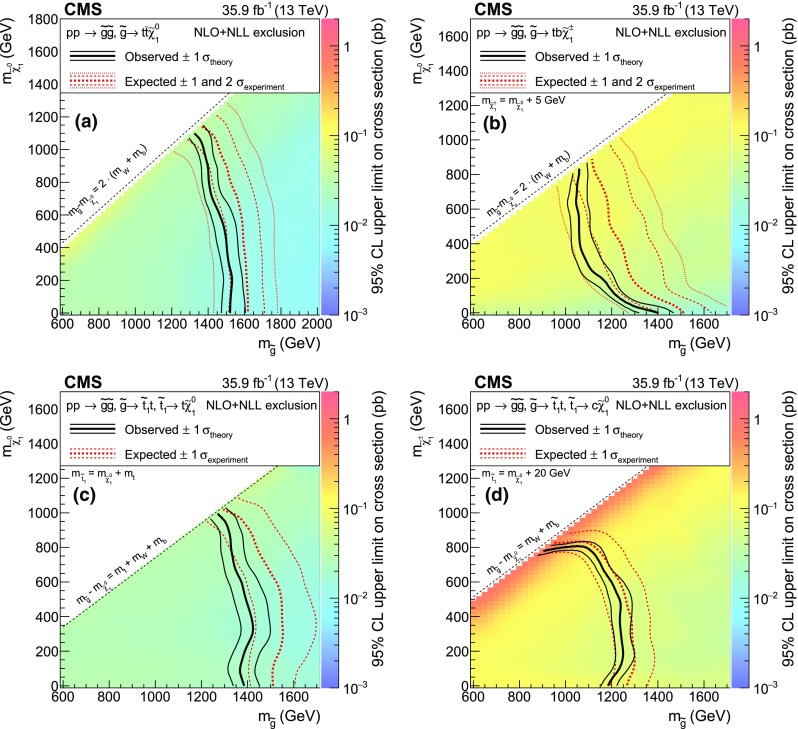
Exclusion regions at 95% CL in the $m_{\widetilde{\chi }^{0}_{1}}$ versus $m_{\widetilde{\mathrm{g}}}$ plane for the $\mathrm{T}1{\mathrm{t} \mathrm{t} \mathrm{t} \mathrm{t}}$  (**a**) and $\mathrm{T}5{\mathrm{t} \mathrm{t} \mathrm{b} \mathrm{b}}\mathrm{W}\mathrm{W}$  (**b**) models, with off-shell third-generation squarks, and the $\mathrm{T}5{\mathrm{t} \mathrm{t} \mathrm{t} \mathrm{t}}$  (**c**) and $\mathrm{T}5{\mathrm{t} \mathrm{t} \mathrm{c} \mathrm{c}}$  (**d**) models, with on-shell third-generation squarks. For the $\mathrm{T}5{\mathrm{t} \mathrm{t} \mathrm{b} \mathrm{b}}\mathrm{W}\mathrm{W}$ model, $m_{\widetilde{\chi }^\pm _{1}} = m_{\widetilde{\chi }^{0}_{1}} + 5\,\text {GeV}$, for the $\mathrm{T}5{\mathrm{t} \mathrm{t} \mathrm{t} \mathrm{t}}$ model, $m_{\widetilde{\mathrm{t}}} - m_{\widetilde{\chi }^{0}_{1}} = m_{\mathrm{t}}$, and for the $\mathrm{T}5{\mathrm{t} \mathrm{t} \mathrm{c} \mathrm{c}}$ model, $m_{\widetilde{\mathrm{t}}} - m_{\widetilde{\chi }^{0}_{1}} = 20\,\text {GeV}$ and the decay proceeds through $\widetilde{\mathrm{t}} \rightarrow \mathrm{c} \widetilde{\chi }^{0}_{1}$. The *right-hand side color scale* indicates the excluded cross section values for a given point in the SUSY particle mass plane. The *solid*, *black curves* represent the observed exclusion limits assuming the NLO+NLL cross sections [46–51] (*thick line*), or their variations of ±1 standard deviation (*thin lines*). The *dashed*, *red curves* show the expected limits with the corresponding ±1 and ±2 standard deviation experimental uncertainties. Excluded regions are to the left and below the limit curves

**Fig. 6:**
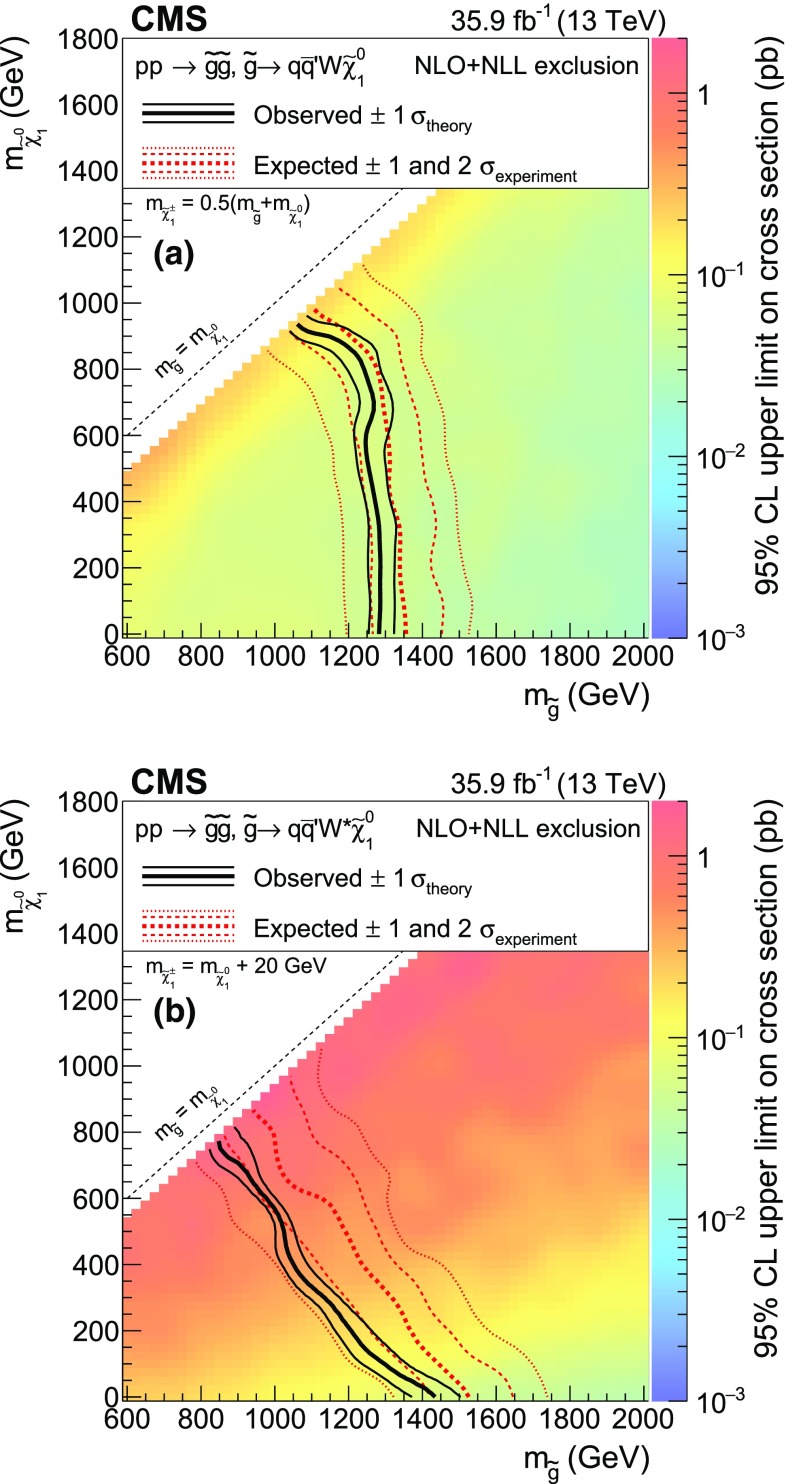
Exclusion regions at 95% CL in the plane of $m_{\widetilde{\chi }^{0}_{1}}$ versus $m_{\widetilde{\mathrm{g}}}$ for the $\mathrm{T}5{\mathrm{q} \mathrm{q} \mathrm{q} \mathrm{q}}\mathrm{W}\mathrm{W}$ model with $m_{\widetilde{\chi }^\pm _{1}}=0.5(m_{\widetilde{\mathrm{g}}} + m_{\widetilde{\chi }^{0}_{1}})$ (**a**) and with $m_{\widetilde{\chi }^\pm _{1}} = m_{\widetilde{\chi }^{0}_{1}} + 20\,\text {GeV}$ (**b**). The notations are as in Fig. 5

**Fig. 7:**
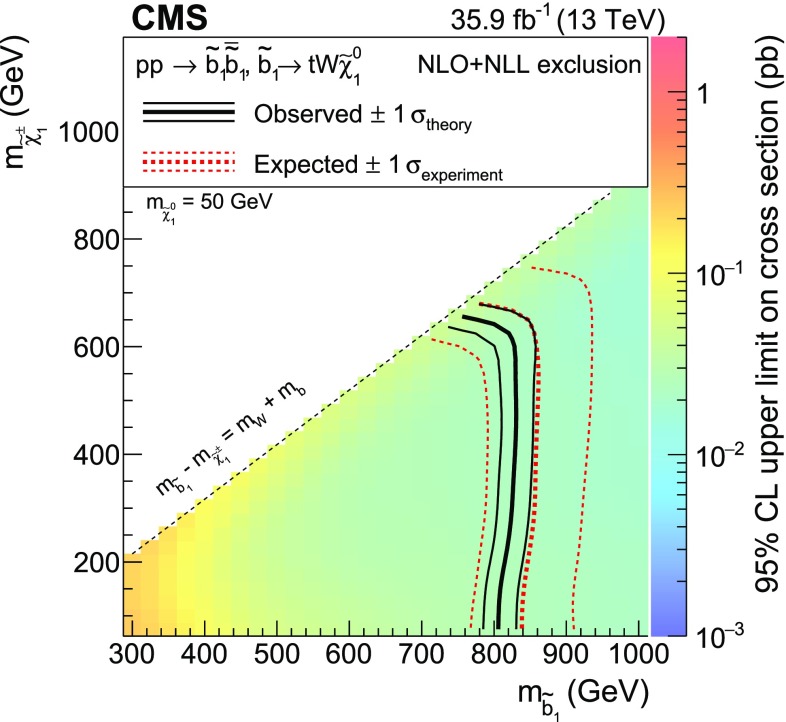
Exclusion regions at 95% CL in the plane of $m_{\widetilde{\chi }^\pm _{1}}$ versus $m_{\widetilde{\mathrm{b}}}$ for the $\mathrm{T}6{\mathrm{t} \mathrm{t}}\mathrm{W}\mathrm{W}$ model with $m_{\widetilde{\chi }^{0}_{1}}=50\,\text {GeV}$. The notations are as in Fig. 5

The observed SUSY cross section limits as a function of the gluino and LSP masses, as well as the observed and expected mass limits for each simplified model, are shown in Fig. 5 for gluino pair production models with each gluino decaying through a chain containing off- or on-shell third-generation squarks. These models, which result in signatures with two or more $\mathrm{b}$ quarks and two or more $\mathrm{W}$ bosons in the final state, are introduced in Sect. 2 as $\mathrm{T}1{\mathrm{t} \mathrm{t} \mathrm{t} \mathrm{t}}$, $\mathrm{T}5{\mathrm{t} \mathrm{t} \mathrm{b} \mathrm{b}}\mathrm{W}\mathrm{W}$, $\mathrm{T}5{\mathrm{t} \mathrm{t} \mathrm{t} \mathrm{t}}$, and $\mathrm{T}5{\mathrm{t} \mathrm{t} \mathrm{c} \mathrm{c}}$. Figure 6 shows the limits for a model of gluino production followed by a decay through off-shell first- or second-generation squarks and a chargino. Two different assumptions are made on the chargino mass, taken to be between that of the gluino and the LSP. These $\mathrm{T}5{\mathrm{q} \mathrm{q} \mathrm{q} \mathrm{q}}\mathrm{W}\mathrm{W}$ models result in no $\mathrm{b}$ quarks and either on-shell or off-shell $\mathrm{W}$ bosons. Bottom squark pair production followed by a decay through a chargino, $\mathrm{T}6{\mathrm{t} \mathrm{t}}\mathrm{W}\mathrm{W}$, resulting in two $\mathrm{b}$ quarks and four $\mathrm{W}$ bosons, is shown in Fig. 7. For all of the models probed, the observed limit agrees well with the expected one, extending the reach of the previous analysis by 200–300$\,\text {GeV}$ and reaching 1.5, 1.1, and 0.83$\,\text {TeV}$ for gluino, LSP, and bottom squark masses, respectively.

**Fig. 8:**
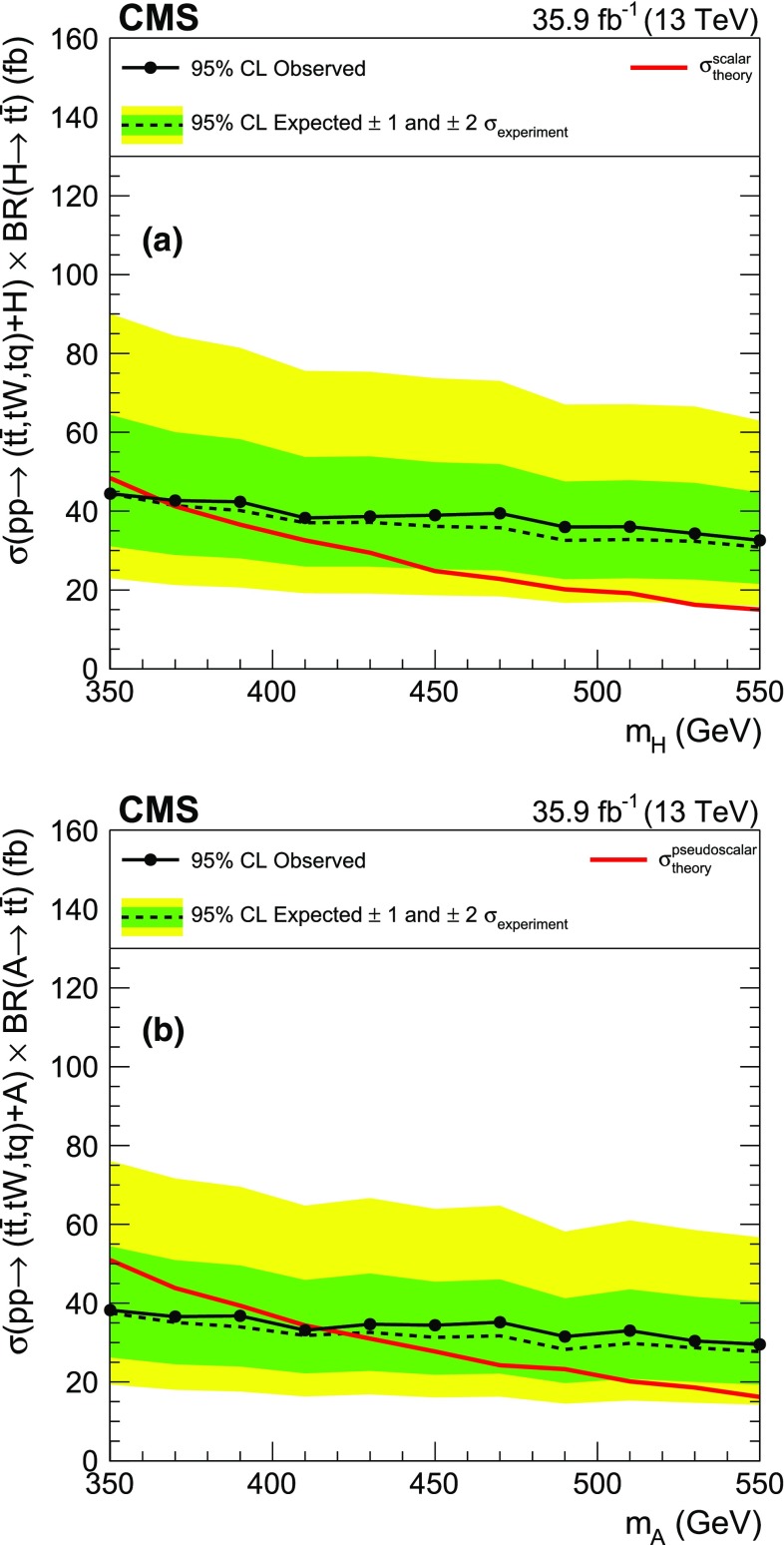
Limits at 95% CL on the production cross section for heavy scalar (**a**) and pseudoscalar (**b**) boson in association to one or two top quarks, followed by its decay to top quarks, as a function of the (pseudo)scalar mass. The *red line* corresponds to the theoretical cross section in the (pseudo)scalar model

The observed and expected cross section limits on the production of a heavy scalar or a pseudoscalar boson in association with one or two top quarks, followed by its decay to top quarks, are shown in Fig. 8. The limits are compared with the total cross section of the processes described in Sect. 2. The observed limit, which agrees well with the expected one, excludes scalar (pseudoscalar) masses up to 360 (410)$\,\text {GeV}$.

The SM four top quark production, $\mathrm{p}\mathrm{p}\rightarrow \mathrm{t}\overline{\mathrm{t}} \mathrm{t}\overline{\mathrm{t}}$, is normally included among the rare SM backgrounds. When treating this process as signal, its observed (expected) cross section limit is determined to be 42 ($27^{+13}_{-8}$)$\,\text {fb}$ at 95% CL, to be compared to the SM expectation of $9.2^{+2.9}_{-2.4}$ $\,\text {fb}$ [33]. This is a significant improvement with respect to the observed (expected) limits obtained in the previous version of this analysis, 119 ($102^{+57}_{-35}$)$\,\text {fb}$ [24], as well as the combination of those results with results from single-lepton and opposite-sign dilepton final states, 69 ($71^{+38}_{-24}$)$\,\text {fb}$  [71].

The results of the search are also used to set a limit on the production cross section for SS top quark pairs, $\sigma (\mathrm{p}\mathrm{p}\rightarrow \mathrm{t} \mathrm{t}) + \sigma (\mathrm{p}\mathrm{p}\rightarrow \overline{\mathrm{t}}\overline{\mathrm{t}})$. The observed (expected) limit, based on the kinematics of a SM $\mathrm{t}\overline{\mathrm{t}}$ sample and determined using the number of b jets distribution in the baseline region, is 1.2 ($0.76^{+0.3}_{-0.2}$)$\,\text {pb}$ at 95% CL, significantly improved with respect to the 1.7 ($1.5^{+0.7}_{-0.4}$)$\,\text {pb}$ observed (expected) limit of the previous analysis [24].

### Model-independent limits and additional results

The yields and background predictions can be used to test additional BSM physics scenarios. To facilitate such reinterpretations, we provide limits on the number of SS dilepton pairs as a function of the $E_{\mathrm{T}}^{\text {miss}}$ and $H_{\mathrm{T}}$ thresholds in the kinematic tails, as well as results from a smaller number of inclusive and exclusive signal regions.

**Fig. 9:**
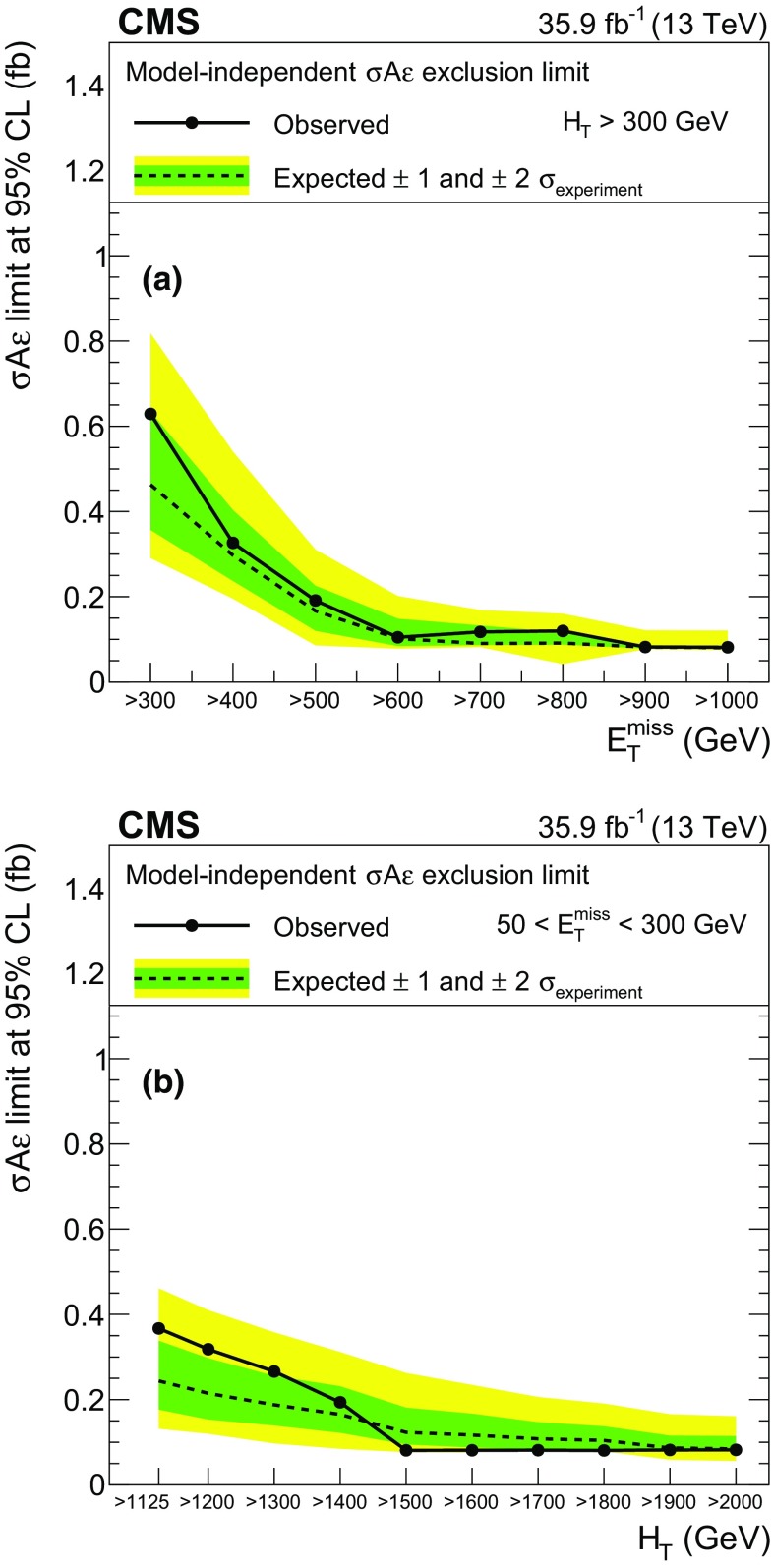
Limits on the product of cross section, detector acceptance, and selection efficiency, $\sigma \! \mathcal {A} \epsilon$, for the production of an SS dilepton pair as a function of the $E_{\mathrm{T}}^{\text {miss}}$  (**a**) and of $H_{\mathrm{T}}$  (**b**) thresholds

The $E_{\mathrm{T}}^{\text {miss}}$ and $H_{\mathrm{T}}$ limits are based on combining HH tail SRs, specifically SR42–45 for high $E_{\mathrm{T}}^{\text {miss}}$ and SR46–51 for high $H_{\mathrm{T}}$, and employing the CL$_\mathrm{s}$ criterion without the asymptotic formulation as a function of the minimum threshold of each kinematic variable. These limits are presented in Fig. 9 in terms of $\sigma \! \mathcal {A} \epsilon$, the product of cross section, detector acceptance, and selection efficiency. Where no events are observed, the observed and expected limits reach 0.1$\,\text {fb}$, to be compared with a limit of 1.3$\,\text {fb}$ obtained in the previous analysis [24].

Results are also provided in Table 7 for a small number of inclusive signal regions, designed based on different topologies and a small number of expected background events. The background expectation, the event count, and the expected BSM yield in any one of these regions can be used to constrain BSM hypotheses in a simple way.

**Fig. 10:**
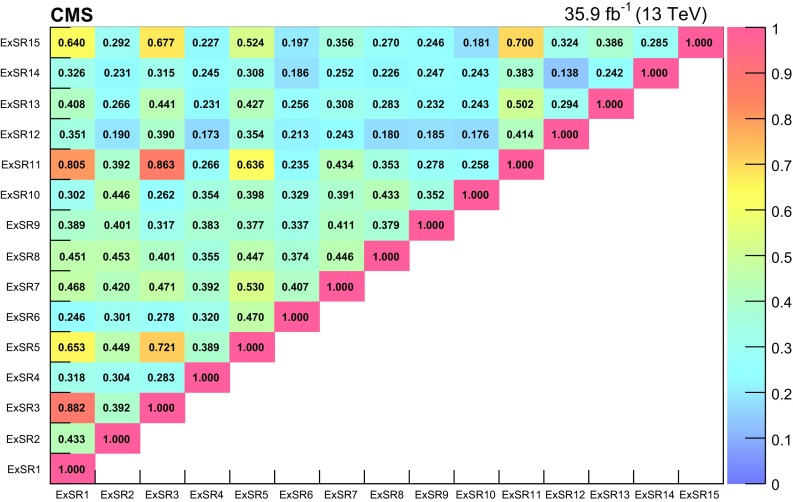
Correlations between the background predictions in the 15 exclusive regions

In addition, we define a small number of exclusive signal regions based on integrating over the standard signal regions. Their definitions, as well as the expected and observed yields, are specified in Table 8, while the correlation matrix for the background predictions in these regions is given in Fig. 10. This information can be used to construct a simplified likelihood for models of new physics, as described in Ref. [72].

**Table 7:** Inclusive SR definitions, expected background yields, and observed yields, as well the observed 95% CL upper limits on the number of signal events contributing to each region. No uncertainty in the signal acceptance is assumed in calculating these limits. A dash (–) means that the selection is not applied

SR	Leptons	$N_\text {jets}$	$N_{\mathrm{b}}$	$H_{\mathrm{T}}$ ($\text {GeV}$ )	$E_{\mathrm{T}}^{\text {miss}}$ ($\text {GeV}$ )	$m_\mathrm{T}^{\text {min}}$ ($\text {GeV}$ )	SM expected	Observed	$N_\text {obs,UL}^\mathrm{95\% CL}$
InSR1	HH	$\ge$2	0	$\ge$1200	$\ge$50	–	4.00 ± 0.79	10	12.35
InSR2		$\ge$2	$\ge$2	$\ge$1100	$\ge$50	–	3.63 ± 0.71	4	5.64
InSR3		$\ge$2	0	–	$\ge$450	–	3.72 ± 0.83	4	5.62
InSR4		$\ge$2	$\ge$2	–	$\ge$300	–	3.32 ± 0.81	6	8.08
InSR5		$\ge$2	0	–	$\ge$250	$\ge$120	1.68 ± 0.44	2	4.46
InSR6		$\ge$2	$\ge$2	–	$\ge$150	$\ge$120	3.82 ± 0.76	7	9.06
InSR7		$\ge$2	0	$\ge$900	$\ge$200	–	5.6 ± 1.1	10	10.98
InSR8		$\ge$2	$\ge$2	$\ge$900	$\ge$200	–	5.8 ± 1.3	9	9.77
InSR9		$\ge$7	–	–	$\ge$50	–	10.1 ± 2.7	9	7.39
InSR10		$\ge$4	–	–	$\ge$50	$\ge$120	15.2 ± 3.5	22	16.73
InSR11		$\ge$2	$\ge$3	–	$\ge$50	–	13.3 ± 3.4	17	13.63
InSR12	LL	$\ge$2	0	$\ge$700	$\ge$50	–	3.6 ± 2.5	3	4.91
InSR13		$\ge$2	–	–	$\ge$200	–	4.9 ± 2.9	10	11.76
InSR14		$\ge$5	–	–	$\ge$50	–	7.3 ± 5.5	6	6.37
InSR15		$\ge$2	$\ge$3	–	$\ge$50	–	1.06 ± 0.99	0	2.31

**Table 8:** Exclusive SR definitions, expected background yields, and observed yields. A dash (–) means that the selection is not applied

SR	Leptons	$N_\text {jets}$	$N_{\mathrm{b}}$	$E_{\mathrm{T}}^{\text {miss}}$ ($\text {GeV}$ )	$H_{\mathrm{T}}$ ($\text {GeV}$ )	$m_\mathrm{T}^{\text {min}}$ ($\text {GeV}$ )	SM expected	Observed
ExSR1	HH	$\ge$2	0	50–300	<1125	<120 for $H_{\mathrm{T}} >300$	700 ± 130	685
ExSR2		$\ge$2	0	50–300	300–1125	$\ge$120	11.0 ± 2.2	11
ExSR3		$\ge$2	1	50–300	<1125	<120 for $H_{\mathrm{T}} >300$	477 ± 120	482
ExSR4		$\ge$2	1	50–300	300–1125	$\ge$120	8.4 ± 3.5	8
ExSR5		$\ge$2	2	50–300	<1125	<120 for $H_{\mathrm{T}} >300$	137 ± 25	152
ExSR6		$\ge$2	2	50–300	300–1125	$\ge$120	4.9 ± 1.2	8
ExSR7		$\ge$2	$\ge$3	50–300	<1125	<120 for $H_{\mathrm{T}} >300$	11.6 ± 3.1	10
ExSR8		$\ge$2	$\ge$3	50–300	300–1125	$\ge$120	0.8 ± 0.24	3
ExSR9		$\ge$2	–	$\ge$300	$\ge$300	–	25.7 ± 5.4	31
ExSR10		$\ge$2	–	50–300	$\ge$1125	–	10.1 ± 2.2	14
ExSR11	HL	$\ge$2	–	50–300	<1125	<120	1070 ± 250	1167
ExSR12		$\ge$2	–	50–300	<1125	$\ge$120	1.33 ± 0.46	1
ExSR13		$\ge$2	–	$\ge$300	$\ge$300	–	9.9 ± 2.5	12
ExSR14		$\ge$2	–	50–300	$\ge$1125	–	4.7 ± 1.8	8
ExSR15	LL	$\ge$2	–	$\ge$50	$\ge$300	–	37 ± 12	43

## Summary

A sample of same-sign dilepton events produced in proton–proton collisions at 13$\,\text {TeV}$, corresponding to an integrated luminosity of 35.9$\,\text {fb}^{-\text {1}}$, has been studied to search for manifestations of physics beyond the standard model. The data are found to be consistent with the standard model expectations, and no excess event yield is observed. The results are interpreted as limits at 95% confidence level on cross sections for the production of new particles in simplified supersymmetric models. Using calculations for these cross sections as functions of particle masses, the limits are turned into lower mass limits that are as high as 1500$\,\text {GeV}$ for gluinos and 830$\,\text {GeV}$ for bottom squarks, depending on the details of the model. Limits are also provided on the production of heavy scalar (excluding the mass range 350–360$\,\text {GeV}$) and pseudoscalar (350–410$\,\text {GeV}$) bosons decaying to top quarks in the context of two Higgs doublet models, as well as on same-sign top quark pair production, and the standard model production of four top quarks. Finally, to facilitate further interpretations of the search, model-independent limits are provided as a function of $H_{\mathrm{T}}$ and $E_{\mathrm{T}}^{\text {miss}}$, together with the background prediction and data yields in a smaller set of signal regions.
